# Two-Stage Priming of Allogeneic Natural Killer Cells for the Treatment of Patients with Acute Myeloid Leukemia: A Phase I Trial

**DOI:** 10.1371/journal.pone.0123416

**Published:** 2015-06-10

**Authors:** Panagiotis D. Kottaridis, Janet North, Maria Tsirogianni, Chloe Marden, Edward R. Samuel, Sam Jide-Banwo, Sarah Grace, Mark W. Lowdell

**Affiliations:** Department of Haematology, Royal Free Hospital, UCL Medical School, Rowland Hill Street, London, NW3 2PF, United Kingdom; Cardiff University, UNITED KINGDOM

## Abstract

Human Natural Killer (NK) cells require at least two signals to trigger tumor cell lysis. Absence of ligands providing either signal 1 or 2 provides NK resistance. We manufactured a lysate of a tumour cell line which provides signal 1 to resting NK cells without signal 2. The tumor-primed NK cells (TpNK) lyse NK resistant Acute Myeloid Leukemia (AML) blasts expressing signal 2 ligands. We conducted a clinical trial to determine the toxicity of TpNK cell infusions from haploidentical donors. 15 patients with high risk AML were screened, 13 enrolled and 7 patients treated. The remaining 6 either failed to respond to re-induction chemotherapy or the donor refused to undergo peripheral blood apheresis. The conditioning consisted of fludarabine and total body irradiation. This was the first UK trial of a cell therapy regulated as a medicine. The complexity of Good Clinical Practice compliance was underestimated and led to failures requiring retrospective independent data review. The lessons learned are an important aspect of this report. There was no evidence of infusional toxicity. Profound myelosuppression was seen in the majority (median neutrophil recovery day 55). At six months follow-up, three patients treated in Complete Remission (CR) remained in remission, one patient infused in Partial Remission had achieved CR1, two had relapsed and one had died. One year post-treatment one patient remained in CR. Four patients remained in CR after treatment for longer than their most recent previous CR. During the 2 year follow-up six of seven patients died; median overall survival was 400 days post infusion (range 141–910). This is the first clinical trial of an NK therapy in the absence of IL-2 or other cytokine support. The HLA-mismatched NK cells survived and expanded in vivo without on-going host immunosuppression and appeared to exert an anti-leukemia effect in 4/7 patients treated.

**Trial Registration:**

ISRCTN trial registry ISRCTN11950134

## Introduction

Acute Myeloid Leukemia (AML) is rarely cured by chemotherapy alone and even high dose therapy with allogeneic transplantation is associated with poor overall survival in patients with adverse prognostic factors as defined by cytogenetic, molecular and other markers. Furthermore, it is only available to those with suitably matched donors and is associated with unacceptable toxicity in elderly patients or in patients with co-morbidities. Therefore, alternative modalities are required which can potentially be applied in all age groups with minimal toxicity and maximum clinical efficacy. In this context, different approaches using immunotherapy or immunomodulation have been tried either as part of induction, during consolidation and less often as maintenance in the treatment of AML.

NK cells play a significant role in the autologous immune response to solid tumors [1] and haematological malignancies such as myeloma [2] and AML [3,4] but their use as adoptive immunotherapy has led rarely to success. In AML, where the malignant blast cells express high levels of MHC class I antigens [5] which may inhibit autologous NK cell function, attention has focussed on use of HLA-mismatched NK cells from haploidentical donors [6, 7, 8, 9]. However, resting human NK cells require two signals to initiate cytokine secretion and cytotoxicity [10] and AML blasts frequently lack one or both of the cognate ligands. We identified an NK priming mechanism which is independent of cytokines and is meditated by tumor cells, including the leukemia cell line CTV-1 (DSMZ) [3]. This priming mechanism is meditated through CD2 and is unique in that, unlike cytokine-mediated priming, tumor-primed NK cells retain the primed state even after cryopreservation [11]. Moreover, the tumor-primed NK cells lyse a wide range of NK-resistant tumors including breast and ovarian cancers [3], lymphomas and prostate cancer [11] and primary myeloma cells [2]. The ability to use cryopreserved aliquots of primed, donor NK cells is a fundamental advance in adoptive NK cell therapy as products can be manufactured centrally, cryopreserved to allow confirmation of functional activity, tested to ensure sterility before release and shipped under temperature-controlled conditions to the patient’s bedside for administration. This allows compliance with the European Union, US and Australian medicines regulations which designate these cells as medicines and thus require robust quality control during manufacture and at the time of release.

We have developed a manufacturing process which complies with EU pharmaceutical Good Manufacturing Practice (GMP) and US cGMP and which generates highly purified, dosed aliquots of allogeneic, primed NK cells from related haploidentical donors, which lyse NK-resistant tumor cells. Here we present the data from the first clinical trial of these tumor-primed NK cell suspensions as an investigational medicinal product (IMP).

## Patients and Methods

### Ethics statement

This trial was approved by the UK Medicines & Healthcare products Regulatory Agency and by the institutional Royal Free Hospital NHS Trust Local Research Ethics Committee (EudracT number: 2005-006087-62) and by the Clinical Trials Committee of the UK *Leukaemia Lymphoma Research* which was the principal funding body. All patients and their related HLA-mismatched NK cell donors gave written, informed consent.

The protocol for this trial and supporting TREND checklist are available as supporting information; see S1 Protocol and S1 TREND Checklist.

### Patient eligibility

A phase 1 dose escalation trial was planned for patients with acute myeloid leukemia (AML) excluding APML. Patient eligibility criteria were as follows: 1) Age >60 years in partial remission (PR) after two courses of induction chemotherapy, or in second complete remission (CR2) after reinduction chemotherapy; 2) Age >60 years with poor risk disease using standard MRC criteria in CR or PR after two courses of chemotherapy; 3) age <60 years beyond CR2 who were not suitable for stem cell transplantation with conventional myeloablative or reduced intensity conditioning regimens.

The NK cell donors were HLA-haploidentical blood relatives of patients, aged 18–65 years. HLA typing was determined for patients and donors using high resolution molecular techniques according to our standard hemopoietic stem cell transplant (HSCT) practice.

### Patient Characteristics

**Fig 1:**
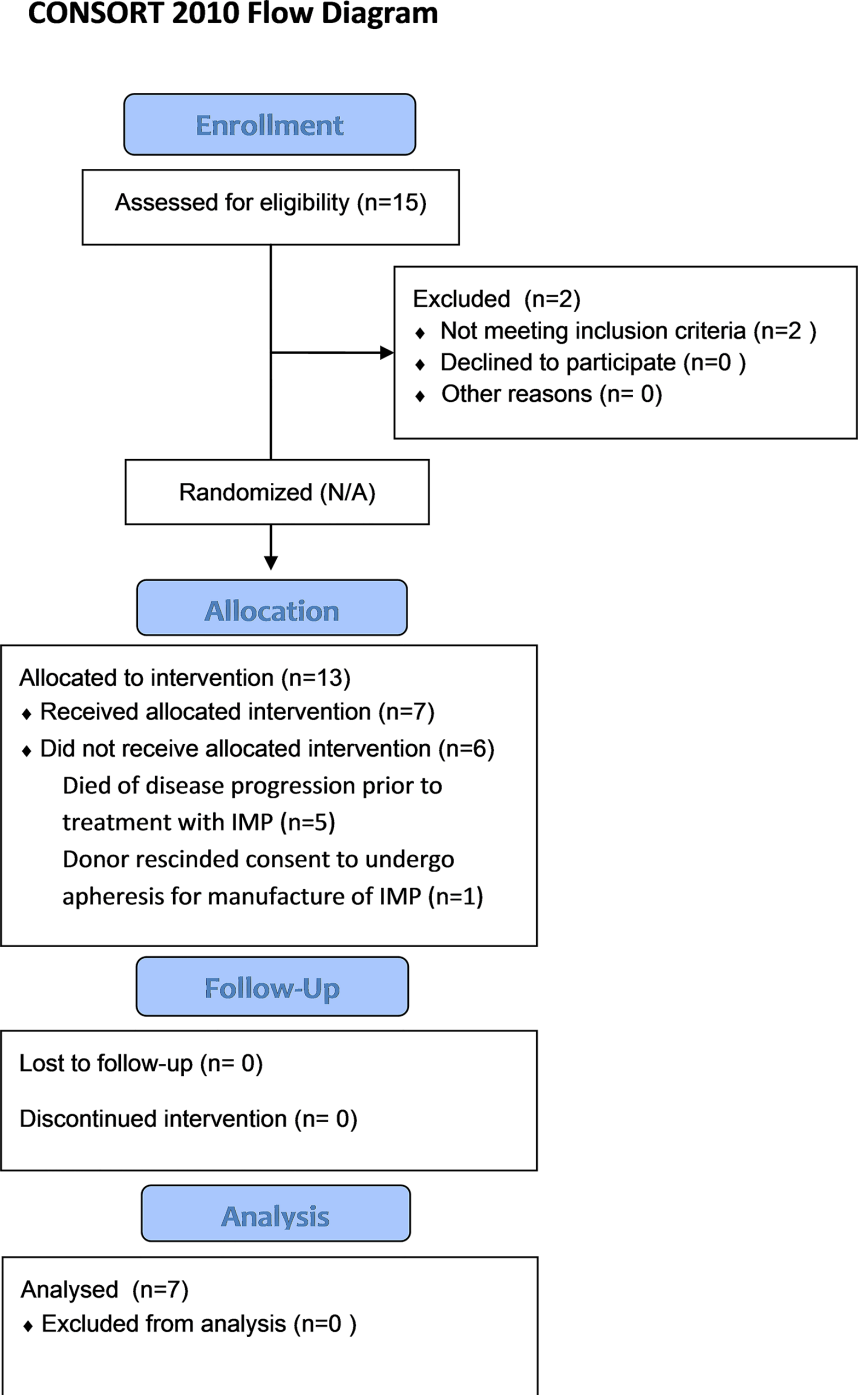
Trial enrollment summary.

Between July 2008 and January 2010, a total of 15 patients with high risk AML were screened; two of whom failed to respond to re-induction chemotherapy and thus failed enrolment criteria. Of the 13 patients enrolled four patients (median age 54 years, range, 22 to 76 years) died of primary refractory/relapsing disease and one patient died of neutropenic sepsis during reinduction chemotherapy before any had received NK cell treatment. One patient was withdrawn from the study as the donor refused to undergo peripheral blood apheresis (Fig 1).

Thus a total of seven patients finally proceeded to receive donor TpNK cell infusion. Characteristics of the evaluable patients are summarized in Table 1. The median age at diagnosis was 65 years (range, 49 to 73 years) and 67 years at treatment. The majority of patients were male (71.4%). Four patients (57.1%) were beyond second-line chemotherapy and three (42.9%) were beyond third-line chemotherapy for relapsing AML, where one patient had undergone autologous hemopoietic stem cell transplantation (HSCT) and two (25%) allogeneic HSCT from HLA matched donors.

**Table 1:** Patient Characteristics.

UPN	sex	Age at diagnosis (years)	Age at treatment(years)	Cytogenetics	1^st^ line chemotherapy	2^nd^ line chemotherapy	3^rd^ line chemotherapy	Allo HSCT	CR 1 duration (days)	CR2 duration (days)	recent CR duration pre- IMP (days)	Disease status at treatment
CTI-01	F	51	54	trisomy 21	DA/My, DA, MACE, MidAC	FLAG, MACE, BuCy, Auto HSCT	HiDAC	no	540	419	30	CR3
CTI-03	M	71	72	N/A	5 azacytidine			no				PR1
CTI-04	M	49	51	t(9;11)	ADE×2, MACE	ICE, BuCy AlloHSCT	ICE	yes	210	270	30	CR3
CTI-07	M	71	71	Complex	DA×2			no			60	CR1
CTI-09	F	73	73	Trisomy 13	DA×1			no			47	CR1
CTI-08	M	65	67	Normal	DA×3,5 azacytidine	FLAG		no	839		62	CR2
CTI-11	M	56	61	Trisomy 9	DA×2,FluCy-AlloHSCT	Rx (auditory meatus MS)	HiDAC	yes	1320	240	90	CR3

Abbreviations: UPN, unique patient number; DA, Daunorubicin + Cytosine Arabinoside; My, Mylotarg; ADE, Cytosine Arabinoside + Daunorubicin + Etoposide; MACE, Amsacrine +Cytosine Arabinoside+Etoposide; MidAC, Mitoxantrone+Cytosine Arabinoside; FLAG, Fludarabine+ Cytosine Arabinoside+G-CCF; BuCy, Busulfan+Cyclophosphamide; ICE, Ifosfamide+Cyclophosphamide+Etoposide; Rx, Radiotherapy; MS, Myeloid Sarcoma; HiDAC, High dose Cytosine Arabinoside; HSCT, Haemopoietic Stem Cell Transplantation; CR, complete remission; PR, partial remission; IMP, Investigational Medical Product; N/A, not available

The Karnofsky performance at treatment was 100% and no patients had co-morbidities. However, one patient had type II diabetes mellitus, one patient suffered from chronic Hepatitis C virus infection on antivirals (lamivudine) and one had undergone prostatectomy due to prostate cancer 11 years prior to his diagnosis of leukemia.

The disease status at the time of the IMP infusion was as follows: Two patients (28.6%) were in first complete remission (CR1) with poor risk disease, one patient (14.3%) in second (CR2), three patients (42.9%) in third (CR3) and one patient was in first partial remission (PR1) with fewer than 25% blasts in the marrow. The median duration of first complete remission period for patients who were beyond first relapse was 689.5 days (range 210–1320 days), the median duration of second complete remission period for those who were beyond second relapse was 270 days (range 240–419 days). Median follow up time was 433 days (range 148 to 1180 days).

### Conditioning regimen and supportive care

**Fig 2:**
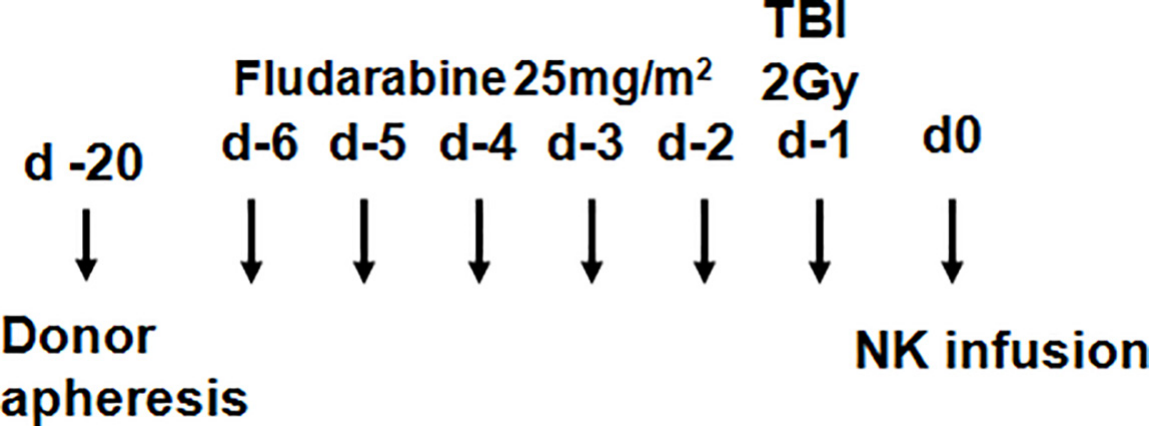
Patient conditioning regimen.

The conditioning regimen for patients comprised of fludarabine 25mg/m^2^ for 5 days and Total Body Irradiation (TBI) as a single dose of 2 Gy (Fig 2). However, the protocol was amended after the first patient’s treatment due to prolonged cytopenia and the dose of fludarabine was reduced to 3 days, while the TBI remained unchanged.

Anti-infective prophylaxis was initiated at commencement of the conditioning and consisted of acyclovir 200mg bd and itraconazole 200mg iv as per institutional guidelines for patients undergoing HSCT.

### Trial Protocol

After identification of suitability, patients were assessed and enrolled after informed consent. This initiated HLA typing of the patient and one or more consenting, related donors. Where more than one donor was available preference was given to those with HLA mismatch detectable by flow cytometry as described below. Presence or absence of predicted KIR ligand mismatch was random.

The trial was a 3-stage dose escalation protocol (S1 Protocol) in which the first 5 patients would each receive a single dose of 1x10^6^ NK/kg patient body weight. In the absence of dose limiting toxicity the next five patients would receive 5x10^6^ NK/kg and the third cohort would receive 1x10^7^ NK/kg. In all cases the patients would receive <10^4^ T cells/kg patient body weight to prevent graft versus host disease (GvHD) and this was the primary release criterion for the IMP. Treatment of all patients within a cohort was to be separated by at least 7 days.

Dose escalation required approval of the Data Safety Monitoring Committee and could only occur a minimum of 1 month after infusion of the 5^th^ patient of the lower dose cohort. After treatment of the second patient in the first cohort the protocol was amended to allow a second infusion of a single dose of 1x10^6^ cells/kg in the event of relapse, but without additional conditioning chemotherapy.

### Donor apheresis and NK cell generation

All consenting donors underwent a 2 hour apheresis approximately 20 days prior to the infusion and none of them experienced any complications. The median number of mononuclear cells recovered was 117.3x10^8^ of which 49.1x10^8^ were CD56-ve/CD3+ve T cells and 10.05x10^8^ were CD56+ve/CD3-ve NK cells. 6.27x10^8^ were CD56+ve/CD3+ve NKT cells. The donor mononuclear cells fractions were labelled with anti-CD56 paramagnetic microbeads (Miltenyi Biotec UK, Ltd) and positively selected by CliniMACS (Miltenyi Biotech GMBH). The mean purity of CD56+ cells was 97.17% of which over 80% were CD56+ve/CD3-ve NK cells. There was a 3.5 log fold reduction in T cells and all products met the acceptance threshold of <1x10^4^ T cells per Kg patient body weight.

The CD56+ fraction was incubated overnight in X-Vivo10 (BioWhittaker) with a lysate derived from CTV-1 leukemia cells (DSMZ) grown to GMP-compliance in our own laboratories. Briefly, CTV-1 cells maintained in X-Vivo10 supplemented with 5% human serum albumin (Bio Products Laboratory, Elstree, UK) were transferred to 50ml Cryocyte bags (Miltenyi Biotech, Ltd, Bisely, Surrey UK) and frozen overnight at -80°C. Cells were thawed rapidly at 37°C, frozen again at -80°C and thawed for a second time in the presence of DNAse (Pulmozyme, Roche, Welwyn Garden City UK). The resultant cell lysates were centrifuged at 2500 x “g”, resuspended in X-Vivo10 at an interpolated concentration of 2x10^7^ CTV-1 /bag and transferred to storage at -80°C after test aliquots had been removed. Batches of CTV-1 lysate were tested for sterility, Mycoplasma and endotoxin as well as functional ability to prime resting, donor NK cells in vitro. Batches passing the QC assays were released for use in GMP-compliant manufacture of primed NK cells.

After overnight co-incubation of resting donor NK cells with CTV-1 lysate, the lysate was removed by discontinuous density gradient separation and aliquots of NK cells were taken to confirm NK priming in a functional assay. Patient-specific doses were cryopreserved in individual aliquots in nitrogen vapour phase and formally released upon completion of sterility and functional testing. Patient-specific products were dispensed for infusion upon completion of the conditioning treatment and were thawed at the patient bedside for immediate administration.

### Patient immune monitoring

Anticoagulated and clotted peripheral blood samples from patients were taken pre-infusion and at approximately 7, 14, 21, 28, 57, 90 and 180 days post infusion according to the trial protocol and patient assent. Additional samples were taken at later time points in long term surviving patients. Bone marrow aspirates were taken as indicated for clinical management and excess sample material was released for immune monitoring as described in the protocol when available.

**Fig 3:**
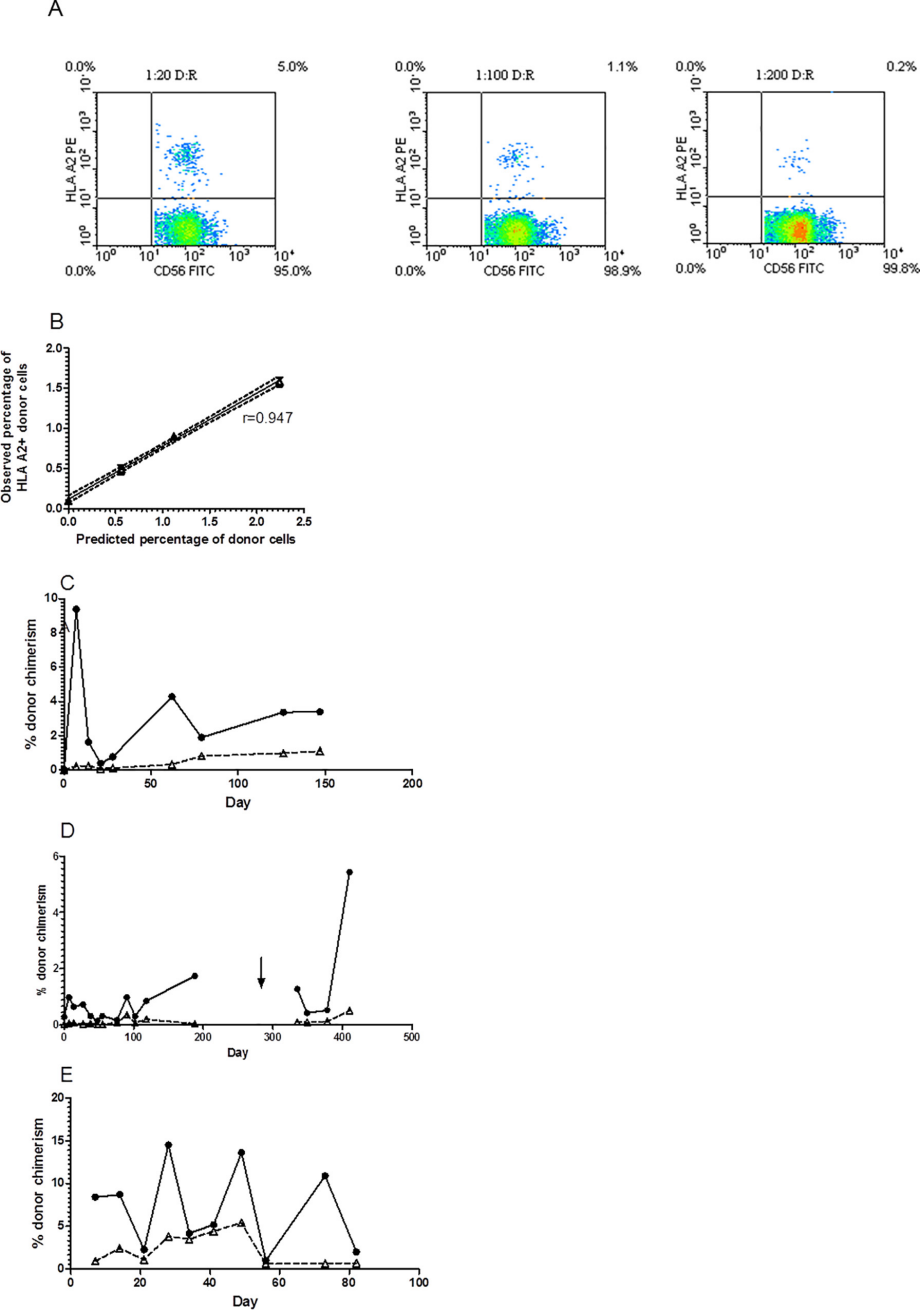
Donor NK chimerism. Donor NK chimerism was measured by flow cytometry using monoclonal antibodies specific for an MHC Class I allele which was restricted to the donor cells. Concomitant labeling with anti-CD3 and anti-CD56 allowed detection of donor T, NKT and NK cells in patients’ peripheral blood and bone marrow. Preliminary experiments confirmed sensitivity (A) and specificity; indeed the method consistently underestimated the dosed proportion of donor cells (B). Three evaluable patients showed consistent presence of low levels of donor NK cells (filled circles and solid line) in peripheral blood (C, D, E). Patient 01 (C) and Patient 03 (D) showed absence of donor T cells (open triangles and dashed line). Patient 04 who suffered profound marrow aplasia and required allogeneic stem cell rescue was the only one to have detectable donor T cells in repeated blood samples (E).

All samples were analysed for T and NK cell subsets and for expression of CD69 as an indicator of *in vivo* cell activation. In patients with sufficient circulating NK cells samples were also tested for the presence of circulating primed NK cells capable of in vitro lysis of NK resistant RAJI cells without additional priming. In patients for whom an informative HLA-mismatch could be detected by a specific anti-MHC class I monoclonal antibody samples were also tested for microchimerism by flow cytometry to determine the proportion of donor cells within the circulating CD56+/CD3- NK and the CD56-/CD3+ T cell pools. Briefly, a minimum of 10^6^ peripheral blood mononuclear cells were labelled with anti-CD3 FITC, anti-CD56 APC and either anti-HLA A2, or anti-HLA A24 or anti-HLA B7 conjugated to PE, depending upon the specific mismatch between donor and recipient and analysed by flow cytometry. The analysis was restricted to cases where the donor cells expressed the specific HLA type which was lacked by the recipient, leading to detection of HLA-expressing donor cells in recipient samples. As shown in Fig 3A and 3B, this flow cytometric assay was pre-validated by “spiking” experiments to a sensitivity of 0.2% donor cells in recipient blood. Briefly, donor NK cells expressing a mismatched MHC class I allele for which a specific monoclonal antibody was available were spiked into a suspension of recipient blood mononuclear cells at dilutions ranging from 1:20 to 1:200. These donor cells were reliably detected with high reproducibility and a significant correlation (coefficient of variance of <10%). The inverse situation where donor cells lacked a specific HLA antigen which was present on recipient cells was not used due to high frequency of “false positive” pre-infusion blood samples.

## Results

### Clinical Outcome

The first patient treated experienced prolonged pancytopenia after infusion of the TpNK dose of 1x10^6^/kg as discussed below in “Adverse Events and Toxicity”. This led to a protocol amendment and a reduction in the intensity of the conditioning regimen for the remaining patients. However, despite this, the following patient in cohort 1 suffered the same pancytopenia and the DSMC decided that an additional patient must be treated at the starting dose of 1x10^6^/kg, making 6 in the first cohort, before a decision on dose escalation could be made. The 4^th^ patient treated also experienced profound and prolonged neutropenia and thrombocytopenia and it was decided that the maximum tolerated dose had be achieved for this patient population and with this conditioning regimen. Dose escalation was halted and all patients treated received a dose of 1x10^6^/kg TpNK.

At six months post IMP infusion, three patients treated in CR remained in remission phase of disease (37.5%), two had relapsed and one had died. One patient who had not achieved CR after conventional induction chemotherapy and was infused at PR1 had achieved CR1 after 50 days from the infusion, He remained in complete remission, relapsing 10 months later. A protocol amendment allowed him to receive a second dose of primed NK cells from the same donor on day +327 but without immunosuppressive conditioning chemotherapy. The HLA-mismatched donor NK cells engrafted without lymphodepleting conditioning and despite complete absence of immunosuppression. He never regained CR but remained stable for a further +203 days after the 2^nd^ infusion while in relapse.

At one year post treatment only one patient remained in disease remission, three more had relapsed but no more deaths has occurred (Table 2). Four patients remained in CR after IMP treatment for longer than their most recent previous CR. All patients finally relapsed. Median time to relapse was 253.5 days post TpNK infusion (range, 58 to 845 days) and the median overall survival was 468.5 days (range, 148 to 1180 days).

**Table 2:** Response after Treatment.

UPN	CR duration post IMP(days)	disease status day +100	disease status day +170	disease status +12 months	Overall survival post- IMP (days) ^c^
CTI-01	845	3^rd^ CR	3^rd^ CR	3^rd^ CR	1180†
CTI-03	250^a^	1^st^ CR	1^st^ CR	1^st^ relapse	530^b^ †
CTI-04	284	3^rd^ CR	3^rd^ CR	3^rd^ relapse	504 †
CTI-07	55	2^nd^relapse	Deceased	deceased	148 days †
CTI-09	141	1^st^ CR	1^st^ relapse	1^st^ relapse	433 days †
CTI-08	116	2^nd^ CR	2^nd^ relapse	2^nd^ relapse	366 days †
CTI-11	352	3^rd^ CR	3^rd^ CR	3^rd^ relapse	414†

Abbreviations: IMP, Investigational Medical Product;

^a^ complete remission was achieved 50 days after the IMP infusion

^b^ the patient received 2^nd^ IMP infusion upon relapse on day+327 and died 203 days after the second infusion

^c^ last follow-up on 01/02/2011

† deceased patients

**Fig 4:**
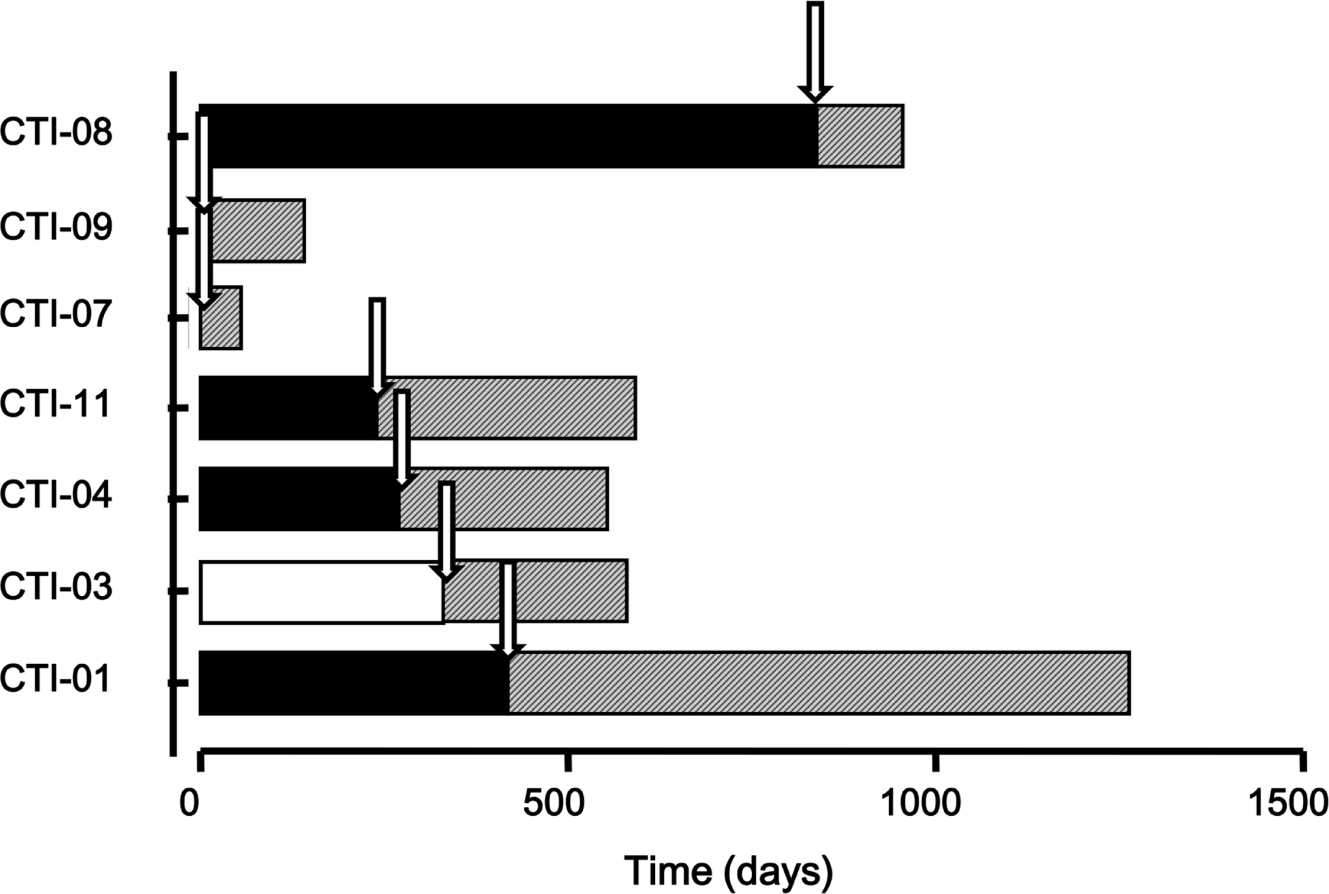
Clinical outcome. Duration of most recent previous CR (black blocks) or PR (white block) prior to ATIMP administration (white arrow) is shown relative to the duration of CR post IMP infusion (gray blocks). Patients 01, 03, 04 and 11 all experienced a longer duration of remission post IMP infusion than their previous CR which was not seen in the other patients.

During the 2 year follow-up period, six out of seven trial patients (86%) (Fig 4) died as did the single patient treated off-trial. The cause of death was AML relapse in five patients, intracranial haemorrhage due to profound thrombocytopenia in one patient and septic shock in the sixth. The median duration of survival after relapse was 174 days (range, 90 to 335 days).

### Donor NK cell chimerism

**Fig 5:**
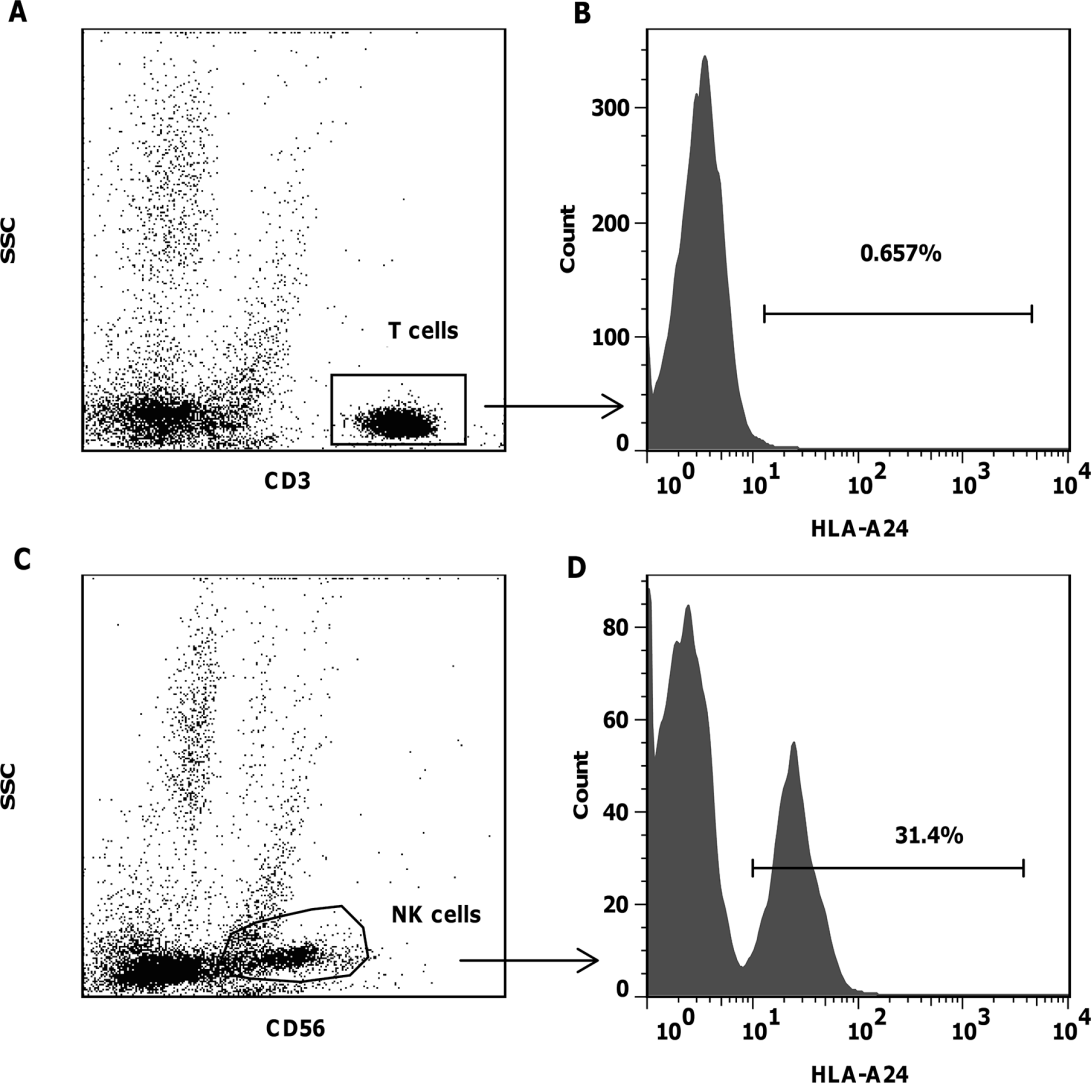
Detection of donor NK cells in bone marrow. A single bone marrow aspirate taken from Patient 03 48 days after receipt of a second infusion of primed donor NK cells showed absence of HLA-A24+ donor T cells (A and B). Donor NK cells (C) expressing HLA-A24 represented almost 1/3 of the total NK cells in the bone marrow (D).

Three of the seven treated patients (CTI-01, 03 and 04) received donor products which were identifiable by flow cytometric chimerism assay. All three patients had detectable donor NK cells in their peripheral blood and/or bone marrow at more than one time point post infusion (Fig 3C, 3D and 3E). The maximum donor NK chimerism in peripheral blood after a single infusion of 1x10^6^ NK/kg ranged from 1.7% in patient 03 to 22.5% in patient 04. The highest achieved in patient 02 being 9.4%. These were all in the absence of detectable donor T cells. Patient 03 received a second infusion after an approved protocol amendment, without additional conditioning. He had attained a maximum peripheral blood NK chimera after the first infusion of only 1.74% and showed an undetectable peripheral blood NK chimerism 7 and 14 days after the second infusion. However, his disease remained controlled and at day +48 a bone marrow aspirate showed an NK donor chimera of over 30% whilst donor T cells remained undetectable (Fig 5). The donor NK cells all expressed the activation antigen CD69 (data not shown).

### In vivo NK cell activation

**Fig 6:**
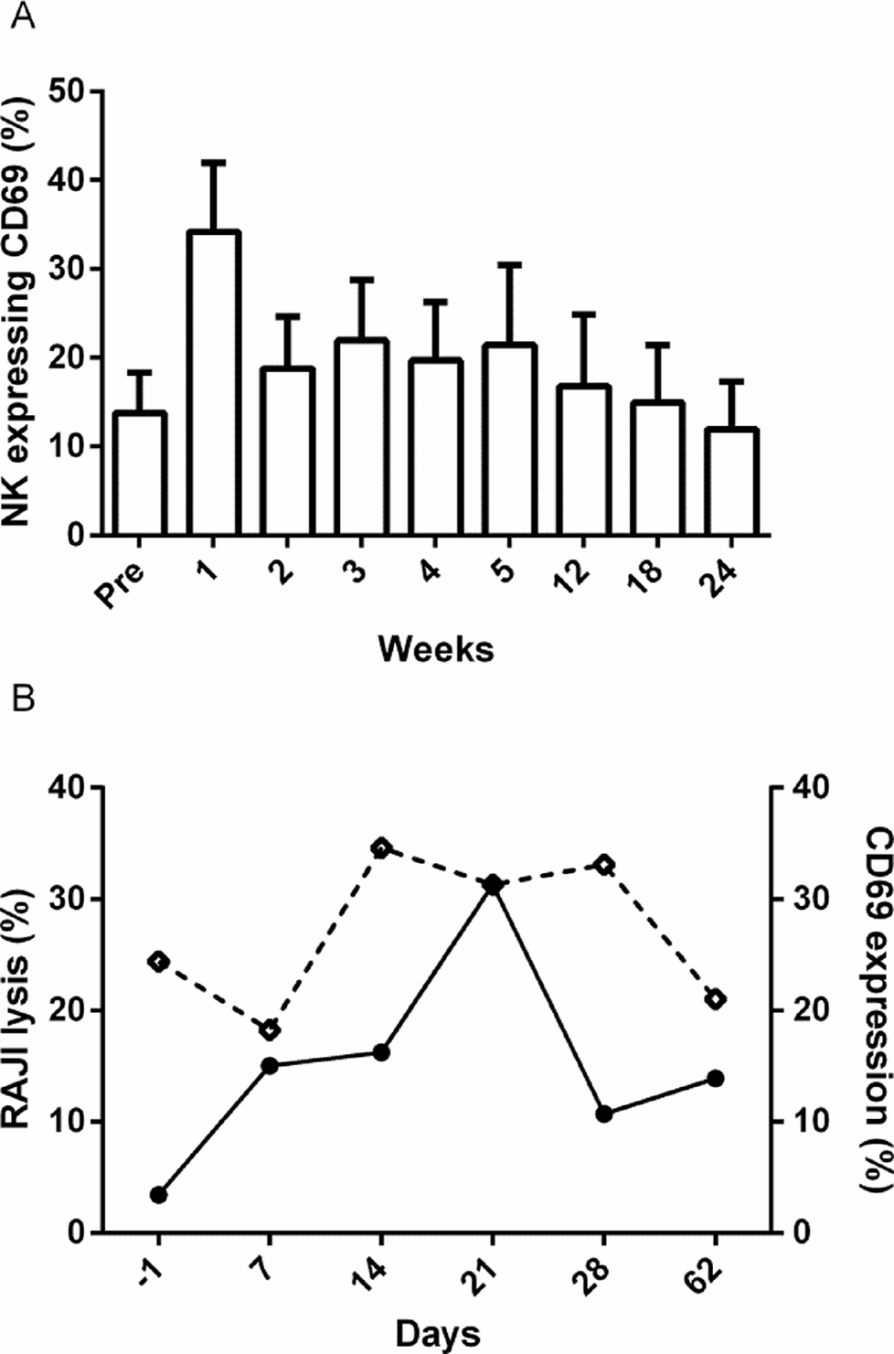
Presence of activated NK cells in peripheral blood. All patients were monitored for the proportions of resting and activated NK cells in their peripheral blood prior to and post primed allogeneic NK cell infusion. NK cell activation in peripheral blood increased within the first two weeks of infusion and did not return to pre-infusion levels until week 12 (A—data shown are mean +/- SD n = 7). A single patient (01) was consistently monitored for the presence of activated NK cells in her peripheral blood which lysed NK-resistant Raji cells in vitro at a fixed effector:target ratio of 5:1 (B—closed circles). The presence of functional primed NK cells closely mirrored the presence of CD69+ NK cells (B—open squares).

Patients were monitored for the presence of activated NK and T cells before and at multiple time points after infusion of the tumor primed NK cell product. Five of six evaluable patients showed the presence of circulating primed NK cells as measured by their ability to lyse the NK-resistant cell line RAJI in an in vitro killing assay. Six of seven evaluable patients showed an increase in the proportion of activated NK cells in their circulation after infusion (Fig 6A). The one patient (03) who did not show an expansion of circulating CD69+ NK cells was the only individual who showed high endogenous activation of autologous NK cells (33.4% CD69+) immediately following conditioning chemotherapy and prior to TBI. The presence of circulating primed NK cells able to lyse RAJI cells coincided with the presence of CD69+ve NK cells in the peripheral blood and was only apparent after infusion of primed donor NK (Fig 6A).

### Adverse events and Toxicity

All seven treated patients were assessable for toxicity post infusion of IMP. All serious adverse reactions (SAR) that were probably or definitely attributed to treatment are listed in Table 3. The median duration of hospitalization post infusion was 31 days (range, 5 to 97 days). Both the conditioning regimen as well as the IMP infusion was tolerated well as none of the patients experienced any infusion related toxicity. None of the patients developed any evidence of GVHD in any organs, however, in the view of cytopenias, bone marrow specific GVHD cannot be excluded.

**Table 3:** Severe Adverse Reactions (SAR).

SAR	No. of Patients N = 7	%
Neutropenia	7	100
Neutropenic fever	7	100
Infection Pneumonia	5	71.4
Probable Fungal pneumonia	3	42.9
Virus infection	2	28.6
Neutropenic sepsis	5	71.4
ITU admission	2	28.6
Platelet reaction	1	14.3
Bone Marrow Suppression	7	100
Aplastic Anaemia	6	85.7
Elevated transaminases	1	14.3
Elevated U, Cr	2	28.6

Abbreviations: ITU, Intensive Care Unit

Hematological toxicity as evidenced by profound myelosuppression was observed in 6 of 7 patients. According to the protocol aplastic anaemia was defined as unexpected neutrophils count <0.5×10^9^/l with Hb<8g/l and platelets <20×10^9^ /l, for more than 28 days. The median time interval to neutrophil recovery (defined as absolute granulocyte count >0.5 ×10^9^/l) was 55 days (range, 19 to 101 days). Platelet recovery (defined as absolute platelet count >50×10^9^/l) was achieved only in four patients (50%) with a median time of 59 days (range, 31 to 114 days). Two patients achieved absolute platelet count >25×10^9^/l after a median period of 64 days (range, 44 to 289 days) with platelet count never exceeding 50×10^9^/l, while one patient remained thrombocytopenic (<25×10^9^/l) throughout the whole follow-up period. The two patients (04, 11) who experienced the longest duration of bone marrow suppression and grade 4 neutropenia unresponsive to G-CSF had both previously undergone allogeneic hematopoietic stem cell transplant as part of their routine treatment prior to this trial. Each of these patientsreceived CD34^+^ cell top up from their original HSCT donor after a median period of 74 days post IMP infusion. This hematopoietic rescue was not within the trial protocol but resolved the neutropaenia in both cases (Table 4).

**Table 4:** Neutropenias.

Patient	Day Neutrophil <0.5	Day Neutrophil >0.5
CTI-01	+8	+59
CTI-03	+5	+40
**CTI-04**	**+18**	**+101**
CTI-07	+4	+19
CTI-09	+2	+51
CTI-08	+15	+43
**CTI-11**	**+15**	**+93**

Subjects in **BOLD** were prior recipients of allogeneic HSCT and received allogeneic CD34+ cell infusions to resolve the grade 4 neutropenias.

The assessment of individual patient’s initial transfusion requirements was continued for a median duration of 84 days (range, 11 to 170 days), and the median number of RBC and PLT units transfused were 16 (range, 2 to 26) and 13 (range, 1 to 35) respectively. Six patients were treated with G-CSF on attending physician’s preference.

Almost all of the patients developed at least one episode of neutropenic fever. All patients were hospitalized for infections and two patients were admitted to the intensive treatment unit (ITU) due to neutropenic sepsis. Among documented infections, five patients developed pneumonia and three had septicaemia. Probable fungal infection was diagnosed in three out of the five patients who developed pneumonia. Hepatic toxicity defined as elevated ALT by>×2 ULN was noted in only one patient while two patients presented elevated urea and creatinine and the values were normalized after a period of 140 days, 42 and 140 days after infusion respectively.

Patient 04 was the third patient treated and the first to experience prolonged and severe neutropenia after the reduction of the conditioning regimen. He was also the first patient who had previously undergone an HLA-identical sibling donor peripheral blood stem cell transplant. The tumor-primed NK cells he received were manufactured from a second, HLA-mismatched brother and, in response to his post treatment neutropenia, he received a CD34 selected top-up graft from his original matched sibling donor. At this time we tested a cryopreserved sample of the batch of tumor-primed NK cells he had received for their ability to inhibit in vitro myelopoiesis by the HLA-matched sibling CD34+ cells in colony forming assays.

A test vial of the investigational product used in this patient was thawed and tumor-primed NK cells were pre-incubated at an effector:target ratio of 10:1 in vitro with CD34+ donor cells for 4 hours and then the co-culture was resuspended in Methocult and cultured for a further 14 days before analysis of the colony formation. The growth of BFU-E, GFU-M and CFU-GEMM was determined in cultures established with and without the TpNK IMP. As a positive control for CFU inhibition, parallel cultures were established with CD3+ T cells from the same donor.

**Fig 7:**
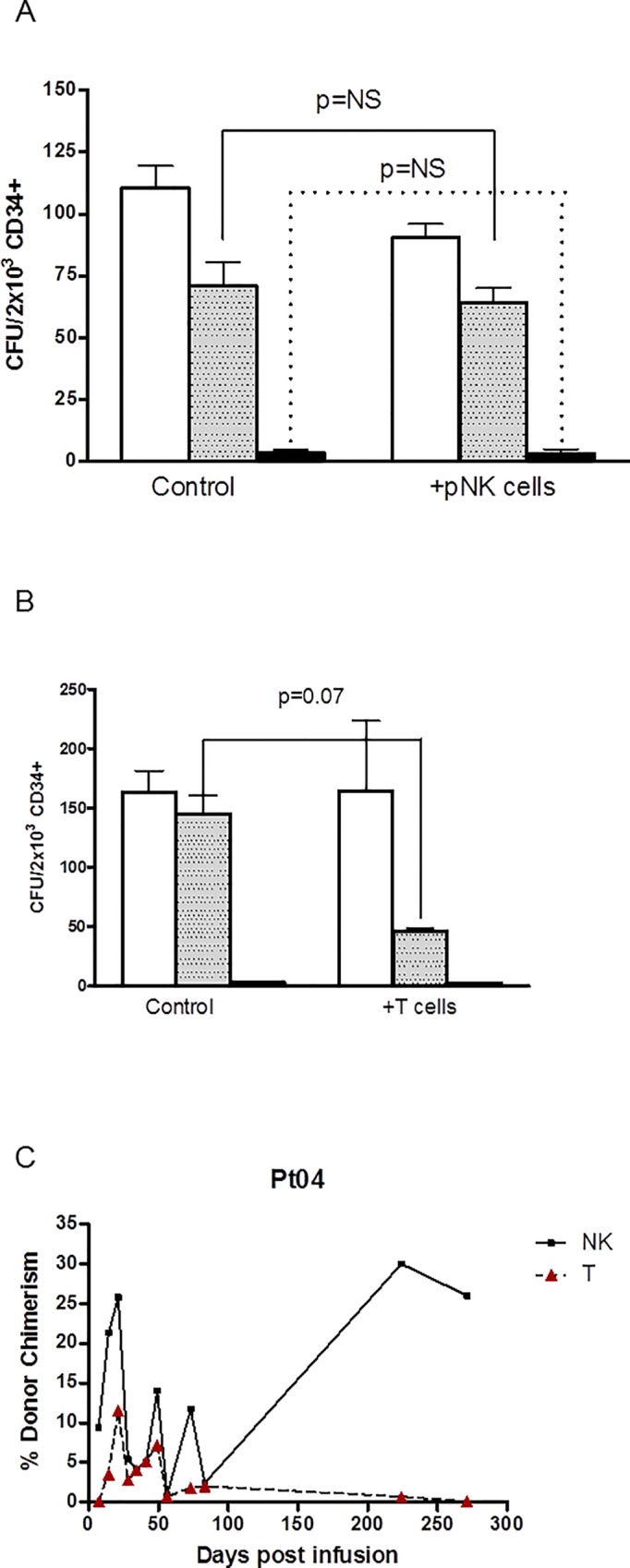
Haploidentical tumor-primed NK cells do not suppress hematopoietic progenitor cells (HPC) in vitro. A—Tumor primed NK cells co-incubated with haploidentical CD34 selected HPC did not suppress BFU-E (white bars), CFU-GM (gray bars) or GFU-GEMM (black bars). B—T cells from the same haploidentical donor substantially inhibited growth of donor CFU-GM (gray bars) C—In vivo donor T cell (Open triangles) and NK (Open Squares) cell chimerism.

As shown in Fig 7A, there was no detectable suppression of CFU-GM, CFU-GEMM or BFU-E in the cultures pre-treated with HLA-mismatched sibling tumor-primed NK cells. In contrast, the donor T cells substantially inhibited GFU-GM whilst the BFU-E and CFU-GEMM were unaffected (Fig 7B). Analysis of microchimerism demonstrated that this patient had an early transient expansion of donor T cells after TpNK infusion which was not seen in other patients who were monitored (fFg. 7c).

### KIR-ligand incompatibility

As shown in Table 5, only two patients received tumor-primed NK infusion in the face of predicted KIR incompatibility. Patient 03 received cells in the face of a predicted host versus donor response and yet showed durable engraftment after both the first and second infusion despite the presence of autologous NK cells at both times. Patient 04 received NK cells with a predicted donor-versus-host reaction and was also the first patient to require Peripheral Blood Stem Cell Transplantation (PBSCT) rescue (the second patient whose aplasia was rescued by PBSCT had no predicted KIR mismatch).

**Table 5:** HLA-KIR mismatch.

PATIENT	Donor	Donor	Predicted KIR m/m
		Class I	
		m/m	
**CTI 01**	**Daughter**	**A 0205**	**No M/M**
		**B 4901**	
		**C 0701**	
**CTI 03**	**Daughter**	**A 2402**	**M/M 2DL1**
		**B 3501**	**Host vs Donor**
		**C 0401**	
**CTI 04**	**Brother**	**A 2601**	**M/M 3DL1**
		**B 1401**	**Donor vs Host**
		**C 0802**	
**CTI 07**	**Son**	**A 0301**	**No M/M**
		**B 3501**	
		**C 0401**	
**CTI 09**	**Daughter**	**A 2601**	**No M/M**
		**B 3801**	
		**C 1203**	
**CTI 08**	**Son**	**A 0101**	**No M/M**
		**B 0801**	
		**C 0701**	
**CTI 11**	**Son**	**A 2901**	**No M/M**
		**B 4403**	
		**C 1601**	

## Discussion

The purpose of this study was primarily to determine the feasibility and safety of haploidentical NK cells following fludarabine and TBI conditioning in a cohort of patients with high risk AML who were not eligible for curative standard allogeneic stem cell procedures or other conventional modalities.

No infusional side effects were noted, however prolonged cytopenias occurred in all patients and most severely in those who had received a previous allogeneic transplant and for whom rescue with a CD34+ stem cell infusion was required from the original donor.

The cause of the aplasia remains obscure and it might be associated either with the toxicity of the conditioning regimen and especially with the TBI, the NK cells or the combination of both. In fact similar cytopenias have been observed in previous studies, however the regimens were different, possibly milder and the NK cells were activated through concomitant administration of interleukin 2 (IL-2). Miller et al reported for the first time the use of IL-2 stimulated haploidentical NK cells in a cohort of patients with poor prognosis AML in whom high dose cyclophosphamide and fludarabine was used for conditioning [6]. Two patients experienced grade 4 neutropenia and 2 others developed grade 3 neutropenia following cyclophosphamide. In a similar study by the same group in a cohort of patients with recurrent ovarian and breast cancer, 4/5 patients developed grade 4 neutropenia beyond day+28 when 2Gy of TBI was added in a high dose of cyclophosphamide and fludarabine regimen [8]. The median time to neutrophil recovery in the TBI cohort was 32 days as compared to 15 days in the non TBI one (p = 0.014).

Nevertheless the intensity of the regimen might not be the only explanation for the prolonged cytopenias. The NK cell product might play a significant role especially in patients where prolonged donor NK cell chimerism can be demonstrated. This has also been suggested by both studies from Miller et al and Rubnitz et al [9]. Miller et al [6] treated a patient with relapsed AML post nonmyeloablative double cord transplantation with cyclophosphamide/fludarabine haploidentical NK cells from his sister about 100 days after cord blood transplantation. Full and sustained haploidentical NK-cell engraftment was shown at day +14. As the patient remained neutropenic for 3 weeks GCSF was started and the patient eventually achieved absolute neutrophil count >0.5x10^9^/l six weeks after the NK cell infusion. By that time there was no evidence of cells from the haploidentical donor. In the study by Rubnitz et al [9], all patients but one achieved neutrophil and platelet engraftment by day +21. The only patient with delayed recovery had prolonged NK engraftment with 2% donor NK cells at day+189. This patient had delayed neutrophil and platelet recovery, as well as lymphopenia with an absolute lymphocyte count less than 0.5x10^9^/L until day +189. At day +261 this patient had no detectable donor NK cells and had complete hemopoietic recovery.

Our in vitro assays of tumor-primed NK cell alloreactivity to normal hemopoieitic stem cells in the preclinical phase and during this trial showed no lysis or suppression of normal CD34+ bone marrow cells. However, murine experiments have shown NK activity against normal allogeneic bone marrow is enhanced by low dose TBI which may explain both our results and those of the Minnesota group.

Although this study was aiming to assess toxicity of the haploidentical NK cell product in a cohort of patients with high risk AML, our results indicate that this approach is associated with engraftment; tracking of donor NK cells to the bone marrow and promising clinical outcome. The presence of donor NK cells in the bone marrow whilst undetectable in the peripheral blood is in line with a recent report of rapid marginalisation of donor NK from peripheral blood after administration followed by reappearance in the periphery days later [12]. We did not study the acute dynamics of NK cell engraftment but it is certain that adoptively transferred NK cells can leave the periphery and, as we observed, can marginalise to the bone marrow; presumably in response to the presence of tumor. The fact that only three patients could be monitored for donor NK chimerism prevents any analysis of the presence or level of NK engraftment with respect to outcome although Miller’s group in Minnesota has observed a threshold of donor NK engraftment which is associated with clinical response (Miller—personal communication). Our report here of high frequency of donor NK cells in a bone marrow aspirate whilst undetectable in peripheral blood suggests that chimerism analyses will need to be more thorough than simple peripheral blood monitoring.

It is a common observation in patients with AML who achieve serial remissions after courses of chemotherapy that each subsequent remission is always shorter than its immediate predecessor. Three of our patients experienced longer remissions and one patient who had never previously achieved CR and was in partial remission prior to the NK cell infusion, achieved complete morphological remission with normal peripheral counts for 8 months (Fig 4). Considering the fact that this was a cohort of patients with high risk disease including multiple relapses and even failed previous autologous or allogeneic transplants, additional studies will be required to assess efficacy in patients with less adverse prognostic features.

One of the most remarkable observations was the sustained engraftment of haplomismatched donor NK cells in Patient 03 after a second infusion without further cytoreductive conditioning. This implies that the patient had developed tolerance to the mismatched alleles after the first infusion, raising the intriguing possibility that the initial tumor-primed NK cells may have deleted the host alloreactive T cell clones by a “veto” phenomenon. This has been reported before in murine models but never in a human transplant setting [13]. It raises the possibility of using multiple courses of tumor-primed NK cell therapy to control AML in patients unable to tolerate conventional allogeneic HSCT or who lack a suitable donor.

The study suffered several failures in GCP compliance, mostly due to the complexity of delivery of an allogeneic cell therapy as an advanced therapy investigational medicinal product (ATIMP) in an academic setting. Anecdotally, these failures are not uncommon in such investigator-led cell therapy trials and many were due to inadequacies in the trial protocol despite the fact that it had been externally peer-reviewed by the funding body’s clinical trials committee and the UK Medicines and Healthcare products Regulatory Agency (MHRA) before approval. The allogeneic product required identification, screening and consent of the related NK donor prior to apheresis, product manufacture, patient conditioning and finally treatment. In this group of patients with advanced or poor risk AML the likelihood of disease relapse during this period was underestimated. Furthermore, the relapse or death of patients during this pre-treatment period was not considered to be a “trial related” event by the investigators and yet, according to the protocol, any relapses or deaths during this period should have been reported as a serious adverse event on trial. The protocol could have been written to define more clearly the required reporting period for Severe Adverse Events to exclude events prior to commencement of the conditioning chemotherapy. However, the question of reporting “all deaths on trial” remains a significant problem in studies such as this. Patients with advanced and aggressive disease are at high risk of relapsing and dying prior to treatment with the investigational drug. One might argue that enrolment should be at the last minute such as, in this case, the commencement of the conditioning chemotherapy. This would have maximised the chance of enrolling a full complement of patients who actually received the trial drug and were informative with regard to toxicity and overall safety. However, the fact that the investigational drug is patient specific a requires a clinical intervention to procure the starting material approximately 15 days prior to starting conditioning chemotherapy prevents this under current interpretation of GCP. This trial highlights the need to hold discussions with the GCP regulator and the sponsor over GCP compliance during the trial design to prevent complications later.

Early phase trials of ATIMPs are often restricted to the higher risk patient groups who have failed all lines of conventional treatment. In this trial the number of disease-stage related adverse events, whilst expected, was not predicted in the protocol and thus not listed in the chart of expected adverse events. This highlights the requirement to be very comprehensive in listing the adverse events which are likely to occur in the patient group being studied if they were to remain untreated or to receive best available conventional care. This reduces the number of adverse events requiring expedited reporting and makes GCP compliance considerably less onerous.

It must also be appreciated that recruitment to ATIMP clinical trials is often slower than predicted; this is a very common theme across all fields of cell therapy. It is difficult to justify a clinical trials team employed solely to support a single trial and thus retraining new staff throughout the trial and recording their training and competencies in the trial records is essential. These trials need to be conducted within experienced clinical research facilities with appropriate quality management systems in place.

A final consideration regarding trials of ATIMPs is an ethical consideration unique to such drugs. Unlike any conventional pharmaceutical, patient-specific cell therapy and tissue engineered ATIMPs consist of autologous or allogeneic donor cells as a critical starting material. The procurement of these cells is invariably associated with a clinical procedure which has put the patient or a donor at some degree of risk or, at least, discomfort. Given the paucity of truly informative pre-clinical data on most ATIMPs it is often ethically difficult to justify discarding a product and excluding a patient on the basis of late failure to meet inclusion criteria or some other aspect of a protocol. In our case, the trial was suspended after the seventh patient had received treatment in order to address the non-compliances with GCP discussed above. However, an eighth patient product had been manufactured and a decision was made to release this for treatment on a compassionate basis but to exclude the patient from the trial dataset and the outcome results are excluded from the analysis presented here. Investigators need to consider how to deal with these situations before completing the trial protocol and to ensure that patients or donors who are contributing starting materials are fully aware that the product may not be used in certain circumstances.

In conclusion, this is the first report of adoptive immunotherapy with activated NK cells in the absence of exogenous *in vitro* and *in vivo* IL-2. Furthermore it is the first report of the use of activated NK cells which were cryopreserved prior to administration, quality controlled and released to the patient as a cellular medicine without subsequent, additional activation. This is the first study to demonstrate safety of tumor-primed NK cells in a cohort of patients with high risk AML; albeit with failures in contemporaneous GCP compliance which required retrospective review and reporting. The NK cells can survive *in vivo* even without immunosuppression and exert a potent anti-leukemia effect. In order to assess further the efficacy in adult patients with AML in CR1 a multicenter study is now under IND at multiple US sites.

## Supporting Information

S1 TREND Checklist Non-randomised trial checklist. (PDF)

S1 Protocol Trial Protocol. (PDF)

## References

[1] Imai K, Matsuyama S, Miyake S, Suga K, Nakachi K. Natural cytotoxicity activity of peripheral-blood lymphocytes and cancer incidence: an 11- year follow-up study of a general population. Lancet 2000;356:1795–1799. 11117911 10.1016/S0140-6736(00)03231-1

[2] Katodritou E, North J, Kottaridis P, Verrou E, Gastari V, Chadjiaggelidou C, et al Tumor-primed Natural Killer Cells from Patients with Multiple Myeloma Lyse Autologous, NK-resistant, Bone Marrow-derived Malignant Plasma Cells. Am J Hematology 2001;86:967–973. 10.1002/ajh.22163 21919039

[3] Lowdell MW, Craston R, Samuel D, Wood ME, O’Neil E, Saha V, et al Evidence that continued remission in patients treated for acute leukaemia is dependent upon autologous natural killer cells. Br J Haematology 2001;117:821–827. 10.1046/j.1365-2141.2002.03495.x 12060116

[4] North J, Bakhsh I, Marden C, Pittman H, Addison E, Navarrete C, et al Tumor-primed human natural killer cells lyse NK-resistant tumor targets: evidence for a two-stage process in resting NK cell activation. J Immunology 2007;178:85–94. 17182543 10.4049/jimmunol.178.1.85

[5] Whiteway A, Corbett T, Anderson R, Macdonald I, Prentice HG. Expression of costimulatory molecules on AML blasts may affect duration of first remission. Br J Haematology 2003;120:442–451. 12580958 10.1046/j.1365-2141.2003.04085.x

[6] Miller JS, Soignier Y, Panoskaltis-Mortari A, McNearney SA, Yun GH, Fautsch SK, et al Successful adoptive transfer and in vivo expansion of haploidentical NK cells in patients with cancer. Blood 2005;105:3051–3057. 15632206 10.1182/blood-2004-07-2974

[7] Curti A, Ruggeri L, D’Addio A, Bontadini A, Dan E, Motta MR, et al Successful transfer of alloreactive haploidentical NK cells KIR-ligand mismatched natural killer cells after infusion in elderly high-risk AML patients. Blood 2011;118:3273–9. 10.1182/blood-2011-01-329508 21791425

[8] Geller MA, Cooley S, Judson PL, Ghebre R, Carson LF, Argenta PA, et al A phase II study of allogeneic natural killer cell therapy to treat patients with recurrent ovarian and breast cancer. Cytotherapy 2011;1:98–107. 10.3109/14653249.2010.515582 PMC3760671 20849361

[9] Rubinitz JE, Inaba H, Ribeiro RC, Pounds S, Rooney B, Bell T, et al NKAML: A pilot study to determine the safety and feasibility of haploidentical natural killer cell transplantation in children with acute myeloid leukemia. J Clin Oncology 2010;28:955–959. 10.1200/JCO.2009.24.4590 20085940 PMC2834435

[10] Bryceson YT, March ME, Ljunggren H-G, Long EO. Synergy among receptors on resting NK cells for the activation of natural cytotoxicity and cytokine secretion. Blood 2006;107:159–166. 16150947 10.1182/blood-2005-04-1351 PMC1895346

[11] Sabry M, Tsirogianni M, Bakhsh IA, North J, Sivakumaran J, Giannopoulos K, et al Leukemic priming of resting NK cells is KIR independent but requires CD15-mediated CD2 ligation and natural cytotoxicity receptors. J Immunology 2011;87:6227–6234. 10.4049/jimmunol.1101640 22084431

[12] Brehym C, Huenecke S, Quaiser A, Betz S, Zimmermann O, Soerensen J, et al IL-2 stimulated but not unstimulated NK cells induce selective disappearance of peripheral blood cells: concomitant results to a phase I/II study. PLoS ONE 2011;6:e27351 10.1371/journal.pone.0027351 22096557 PMC3212563

[13] Olsen JA, Leveson-Gower DB, Gill S, Baker J, Beilhack A, Negrin RS. NK cells mediate reduction of GvHD by inhibiting activated, alloreactive T cells while retaining GvT effects. Blood 2010;115:4293–4301. 10.1182/blood-2009-05-222190 20233969 PMC2879101

