# Supplementary material for: Two-Stage Priming of Allogeneic Natural Killer Cells for the Treatment of Patients with Acute Myeloid Leukemia: A Phase I Trial
Source: PLoS One. 2015 Jun 10;10(6):e0123416. doi: 10.1371/journal.pone.0123416 (PMC4465629; doi:10.1371/journal.pone.0123416)
Supplement: S1 Protocol — (PDF) [file pone.0123416.s002.pdf]

Royal Free Hampstead NHS
NHS Trust

Royal Free Hospital
Pond Street
London NW3 2QG

Title:
A phase I trial of allogeneic Tumour-activated natural killer lymphocytes after low dose TBI and fludarabine for the treatment of selected patients with acute myeloid leukaemia.

Short title: TaNK in AML

Protocol Number: TaNK06 Version 1.2 (27th October 2009)

EudracT number: 2005-006087-62

Sponsor: University College London
Gower St
London WC1

Sponsor's representative: Dr Susan Kerrison
UCL/UCLH R&D Office
Tel - 020 7380 9927
e-mail – s.kerrison@ucl.ac.uk

Sponsor's Protocol Code: 07/082

Sponsor's Medical Expert: Professor Stephen Mackinnon
Department of Haematology
University College London
London NW3 2PF
Tel - 020 7472 6860
e-mail – s.mackinnon@ucl.ac.uk

Chief Investigator:
& Authorised signatory Dr. Panagiotis Kottaridis MSc, PhD FRCP, FRCPath
Tel - 020 7794 0500 x35173
e-mail – Panagiotis.kottaridis@royalfree.nhs.uk

Co-investigator:
& Authorised signatory Dr. Mark W. Lowdell PhD, FRCPath
Tel - 020 7830 2183
e-mail – m.lowdell@medsch.ucl.ac.uk

Trial Site: Department of Haematology,
Royal Free & University College Medical School, London
and Royal Free Hospital NHS Trust.
020 7830 2183

IMP production laboratory: Paul O'Gorman Laboratory of Cellular Therapeutics
Royal Free Hospital
London NW3 2QG

Clinical laboratory: Department of Haematology
Royal Free Hospital
London NW3 2QG

Signature Page:

Dr. Panagiotis Kottaridis MSc, PhD FRCP, FRCPath …………………………………….

Dr. Mark W. Lowdell MSc, PhD, FRCPath …………………………………….

CONTENTS:

	Page
Signatories …………………………………………………………………………………	3
Contents ………………………………………………………………………………………	4
History of Amendments ……………………………………………………………………	5
Background Information …………………………………………………………………	6
Trial Objectives and Purpose ……………………………………………………………	8
Trial Design …………………………………………………………………………………	9
Conduct of the Study ………………………………………………………………………	9
Approved Label copy ………………………………………………………………………	9
Selection and Withdrawal of subjects (inclusion / exclusion criteria)……………………….	10
Treatment of Subjects and Donors ………………………………………………………..	12
Registration …………………………………………………………………………	12
Patient pre-infusion chemotherapy …………………………………………………	13
Patient infusion with IMP ……………………………………………………………	13
Assessment of efficacy ……………………………………………………………………..	15
Assessment of safety ………………………………………………………………………	15
Reporting of Serious Adverse Events (SAEs) ……………………………………..	17
Expected Adverse Events Table ……………………………………………………	18
Statistics ………………………………………………………………………………………	19
Direct access to source documents ………………………………………………………..	19
Quality control and Quality assurance …………………………………………………….	19
Ethics …………………………………………………………………………………………	19
Data Handling ………………………………………………………………………………...	20
Financial Indemnity ………………………………………………………………………..	20
Publication Policy ……………………………………………………………………………..	20
Annex 1 – Pre-commencement patient & donor screening ……………………………………	22
Annex 2 – TaNK Trial in Summary …………………………………………………………….	23

Protocol Amendments History Page

Amendment Number & Date	Description of amendment	Date Authorised	
		By MHRA	By Ethics
1 – 13/11/2007	Increased period of observation post TaNK infusion to 6 hours	14/12/2007	15/12/2007
2 – 13/10/2008	Decreased dose of Fludarabine, addition of storage of autologous HPC-A in suitable patients<br>Protocol renumbered v1.1 and -reissued	10/11/2008	

1. Background information

Introduction

Acute myeloid leukaemia (AML) is cured by conventional chemotherapy in fewer than 45% of patients. Patients who relapse after initial chemotherapy but go on to achieve 2nd clinical remission (2CR) are at extreme risk of subsequent relapse and are candidates for allogeneic stem cell transplantation if a HLA-matched donor is available. The toxicity of allogeneic transplants restricts this procedure to physically fit patients. AML is increasingly a disease of the elderly, most of whom are unsuitable for allogeneic transplantation and the prognosis for those who relapse after initial chemotherapy is very poor with a median survival of 7-8 months. We and others have shown that natural killer cells, autologous or allogeneic, can target AML blasts in vitro and in vivo leading to resolution of detectable disease. Furthermore, our group has shown that detectable NK activity to AML cells in patient's peripheral blood is predictive of long term survival post chemotherapy. Most recently our group has described a novel, tumour-specific NK activation protocol which produces activated NK cells capable of high levels of lysis of a broad range of leukaemias.

Natural Killer cells

Natural killer cells (NK) are cytotoxic cells which comprise up to 30% of peripheral blood lymphocytes in normal individuals and up to 50% in AML patients after chemotherapy [1]. In contrast to T cells, NK cells are non HLA-restricted and act in a non-antigen specific manner, their activation is controlled by a balance between stimulatory and inhibitory signals. Our group has been studying the role of NK cells in the control and eradication of AML and other haematological malignancies for over 20 years and we have recently shown that these cells are responsible for the maintenance of remission in patients who respond to chemotherapy alone [2]. The stimulatory ligands on AML cells remain elusive but we and others have made some progress in this area [1,3]. Most recently, NK cells from HLA-mismatched donors have been shown to lyse patient AML cells and to prevent relapse post-transplant [4,5]. Furthermore, allogeneic NK cells can eradicate AML blasts from contaminated stem cell grafts without lysis of normal haematopoietic stem cells [6]. Cytokine-activated allogeneic NK cells have recently been used in the treatment of refractory AML in a phase I trial in which 5 of 19 patients achieved remission and none reported severe adverse events [7]. Thus, accumulating evidence suggests that allogeneic NK cells may play an important role in disease free survival for AML.

Human NK cells require multiple signals to prime them and trigger lysis of tumour cells. Priming signals may be provided by cytokine stimulation such as IL-2, IL-12 and IL-15 and these have been used clinically. However, systemic administration of cytokines is associated with high incidences of severe adverse inflammatory reactions. In contrast, we have shown that human NK cells can be specifically activated to lyse tumour cells by pre-exposure to lysates prepared from a tumour cell line which we believe mirrors the physiological NK cell response to tumours.

The IMP:

"Tumour-activated Natural Killer cells"

Donors will undergo leucopheresis on a single day. Leucopheresis will be performed on a continuous flow cell separator machine. PB mononuclear cells will be collected using a two-arm venous access technique. The anticoagulant used for the procedure is acid citrate dextrose (ACD). Blood flow rates should be maintained at 50 to 60 ml/minute, depending on venous access. The endpoint of each leucopheresis collection will be the processing of **10 to 15 litres of whole blood**.

Upon completion of the apheresis the mononuclear cell products are passed to the LCT for NK cell stimulation and selection. PBMC will be assessed for NK content by flow cytometry, suspended in X-Vivo 10 media at a concentration of $5x10^6$/ml and an appropriate dose of CTV-1 cell-lysate preparation will be added. The cells will be co-cultured overnight at 37^oC / 5% CO_2. Upon completion of the PBMC culture period (Day+1) we will select NK cells by direct isolation with anti-CD56 (Miltenyi Biotec) and CliniMACS. The selected fraction will be analysed for sample NK content, purity and TaNK function. Additional samples will be sent for routine microbiological assessment (LCT RFH/SOP/014/002). A volume of media containing the correct dose of NK cells for the specific patients (**<u>and confirmed to contain fewer that 10^4 CD3+/CD56- T cells /kg</u>**) will be removed and reduced to 10ml by centrifugation, and cryopreserved (following dilution in an equal volume of cryoprotectant to give a final volume of 20ml) at the appropriate patient-specific dose in a Cryocyte bag (Baxter Healthcare).

Safety of Allogeneic Natural Killer cells

The possible role of NK cells in mediating a GvL effect have been discussed. In contrast to allogeneic T cells, it appears that donor NK cells do not mediate GvHD. It is likely that this is due to specific requirements of NK cells for particular adhesion molecules on their target cells which are constitutively present on haematopoietic cells but lacking from most somatic cells. Several hundred allogeneic transplants have been performed in the USA in which the T cell specific antibody OKT3 has been used to T cell deplete the graft. These NK replete, T cell depleted transplants have been associated with low incidence and severity of GvHD. NK cells from HLA-mismatched donors have been shown to mediate GvL without GvHD in vivo [4,5] and our own in vitro studies have supported this [8]. Allogeneic HLA-mismatched NK cells have been shown to purge leukaemia cells from bone marrow whilst sparing normal haematopoietic stem cells [6] and none of the recipients of HLA-mismatched allogeneic NK cells in the recent Miller study [7] showed evidence of GvHD or of suppression of the patients' bone marrow.

Route of administration dosage and dose regimen

Administration will be i.v. in a single 20ml injection as this is the conventional route of administration for cryopreserved therapeutic lymphocyte infusions. The volume of 20ml is in keeping with standard practice for donor lymphocyte infusions which routinely range from 20-50ml.

We plan to infuse a maximum of 10^7 donor tumour-activated NK cells (TaNKs) per kg recipient body weight on the day of completion of chemotherapy and radiotherapy or the following day (the day of infusion will be referred to as "Day 0"). This is the same dose of IL-2 activated NK cells given in a comparable trial in Minnesota and no greater than the dose of NK cells included in a T cell depleted haploidentical haematopoietic stem cell transplant. Assuming an average patient of 70kg this represents a total dose of $7x10^8$ donor NK cells. Patients will receive a single dose.

TaNK will be released to the clinician in response to a signed request as a single dose for a named patient as a cryopreserved product in a maximum volume of 20ml. It will be transported to the ward in a monitored dry-shipper and be thawed by a member of the LCT staff by the patient's bedside immediately prior to infusion. Approximately 10min prior to release of the product (i.e. 30min prior to planned infusion), the patient will receive pre-medication of chlorpheniramine IV and paracetemol. On arrival of the cryopreserved product on the ward the patient details on the bag and accompanying paperwork will be checked by the administering clinician (nurse or physician) and matched to the patient's wrist tag. At least three points of cross-identification will be

confirmed. It will be the responsibility of the LCT staff accompanying the product to rectify any mistakes and these must be recorded as a “non-conformance”.
The clinician will confirm that consent for the treatment has been obtained and will then carry out the baseline observations and ensure that all pre-infusion blood samples have been taken. The TaNK product will be recovered from the Cryocyte bag into a syringe using a minimum of a 21G needle and infused intravenously by slow push over approximately 10min in accordance with standard practice for infusion of allogeneic donor lymphocyte infusions.
At completion of the infusion the clinical staff will monitor and record patient temperature, pulse, blood pressure and respirations. Any abnormalities will be reported to the CI and noted in the CRF and patient’s notes.
The patient will remain on the ward for at least 6 hours post infusion and be observed for reactions. Any reactions observed will be reported to the medical staff and the CI and recorded in the CRF. If the patient experiences nausea (a common reaction to the DMSO in cryopreserved products) anti-emetics may be administered.

Study population
All recipients will have a diagnosis of acute myeloid leukaemia (AML) and be in one of the following subgroups:

Patients aged > 60 years in PR (blasts >5<20% in BM) after 2nd course of induction chemotherapy

Patients aged > 60 years with relapsed AML in CR2 after re-induction chemotherapy

Patients aged > 60 years in PR or CR after 2 courses of chemotherapy with poor risk disease using standard MRC criteria

Patients aged < 60 years beyond CR2 who are not suitable for stem cell transplantation with conventional or reduced intensity conditioning protocols.

2. Trial Objectives & Purpose

Primary Objective - To determine the safety of infusion of allogeneic, tumour-activated NK cells after low dose radiotherapy plus medium dose chemotherapy with respect to acute / chronic GvHD and bone marrow suppression.

Secondary Objectives –

1. To assess the quantitative and qualitative aspects of immune responses to acute myeloid leukaemia (AML) cells in these patients after NK cell infusion.
2. To assess long term survival of donor NK cells in the peripheral circulation of recipients.

3. Trial Design

Primary endpoint – Engraftment of donor NK cells.

An open, non-controlled, non-randomised dose escalating design with cohorts of 5 patients will be employed. Initially 15 adult patients with acute myeloid leukaemia (see entry criteria below) will be entered into the study
All patients will have a HLA-haploidentical related donor.

4. Conduct of the study

Donor TaNK infusion (the IMP):

Approved Label copy

Trial Code – UCL 07/082<br><br>"FOR CLINICAL TRIAL USE ONLY"<br><br>**Allogeneic TaNK cells for i.v. infusion**<br>Batch 01/031/2007<br>SINGLE DOSE (10^7/kg patient body weight)<br><br>Trial Patient Study Number **TaNK CTI 001**<br>(**FOR THIS PATIENT ONLY)**<br><br>Store at or below -135oC and thaw immediately prior to infusion<br>Use by 23rd July 2007<br>PI – Dr Panos Kottaridis; Site: Royal Free Hospital; Sponsor – Royal Free Hospital NHS Trust

On Day 0 the initial 5 patients will receive a single prophylactic infusion of 10^6 NK cells/kg recipient body weight. If fewer than two patients show GvHD >grade 2 in the first cohort of patients the next 5 will receive a dose of $5x10^6$ NK cells /kg recipient body weight and if fewer than 3 patients suffer GvHD >grade 2 in the second cohort a further 5 patients will receive a dose of 10^7 NK/kg recipient body weight. No patient will receive TaNK infusion within 7 days of the previous patient in a single dosing cohort or within 14 days of the final patient treated at a lower dose.
All patients will be monitored for adverse events, leukaemia relapse, donor NK engraftment and immune reconstitution for a further six months.

Early termination of the trial

GvHD or aplasia -

Patients will be monitored for infusional toxicity during infusion and for 6 hours post infusion.
GvHD will be monitored in accordance with current clinical practice in the standard operating procedure of our unit.
Based upon the very low incidence of GvHD in reduced-intensity allogeneic transplant recipients who have been received a similar chemotherapy regimen but without NK cell infusion we have set the following thresholds for termination of the trial -

- If >2 patients of the first 5 show evidence of severe GvHD (>grade II) after infusion of the donor NK cell fraction at a dose of 10^6 cells/kg recipient body weight then the trial will cease.
- If >3 patients of the first 8 show evidence of severe GvHD (>grade II) after infusion of the donor NK cell fraction at a dose of $5x10^6$ cells/kg recipient body weight then the trial will cease
- If >3 patients of the first 12 show evidence of severe GvHD (>grade II) after infusion of the donor NK cell fraction at a dose of 10^7 cells /kg recipient body weight then the trial will cease

Patients will be monitored for treatment-related pancytopaenia by virtue of the regular FBC/Diff sampling described above. Treatment-related pancytopaenia is defined as PMN <$0.5x10^9$/l with unsupported Hb <8g and unsupported platelets <20 of greater than 28 days. Subjects from whom a back-up HPC-A collection is available will receive the product for autologous reconstitution.

If no explanation for the pancytopaenia is apparent the subject's condition will be regarded as aplastic anaemia and the trial will cease accrual. The data will be referred to the Data and Safety Monitoring Committee and a decision made regarding the continuation of the trial.

Graft versus host disease treatment:

Standard clinical criteria and skin biopsy histology will be used to establish the diagnosis and grade its severity according to the Glucksberg criteria. Patients with significant grade II or greater GVHD will be considered for acute treatment with high dose corticosteroids with or without other immunosuppressive agents.

5. Selection and withdrawal of subjects

Patient inclusion criteria:

All recipients will have a diagnosis of acute myeloid leukaemia (AML) and be in one of the following subgroups

Patients aged > 60 years in PR (blasts >5<20% in BM) after 2nd course of induction chemotherapy

Patients aged > 60 years with relapsed AML in CR2 after re-induction chemotherapy

Patients aged > 60 years in PR or CR after 2 courses of chemotherapy with poor risk disease as defined by MRC criteria

Patients aged < 60 years beyond CR2 who are not suitable for stem cell transplantation with conventional or reduced intensity conditioning protocols

Suitable HLA matched or mis-matched related NK cell donor.

Life expectancy >8 weeks and absence of coexisting medical problems that would significantly increase the risk of the chemotherapy procedure in the judgement of the haematology physicians (e.g. poor ejection fraction)

Creatinine < 2x normal and not rising, for at least 2-4 weeks before chemotherapy. If creatinine is elevated, then clearance must be greater then 50 ml/min.

Total bilirubin < 35 umol/l, SGOT < 3x upper limit of normal, and none of these parameters increasing, for at least 2-4 weeks before chemotherapy. Exemptions for reasons of congenital hyperbilirubinemia syndromes or liver enzyme/bilirubin elevations due to disease per se should be cleared with the responsible consultant.

Written informed consent - Signed informed consent for enrolment in this protocol will be obtained from eligible patients by the physician before the start of treatment. At the pre-admission consultation, patients will be fully informed of the purposes and potential risks and benefits of allogeneic transplantation. Patients will have the opportunity to have questions answered to their satisfaction before signing informed consent.

Patient exclusion criteria:

HIV 1-2 seropositive.

Psychiatric, addictive, or any disorder which compromises ability to give true informed consent for participation in this study.

Pregnant or lactating women.

Patients whose life expectancy is severely limited by illness other than for which they are undergoing immunotherapy.

Patients with other active malignancy.

Patients with known physical or religious sensitivity or prior exposure to murine and/or ovine proteins.

Donor inclusion criteria:

HLA- haploidentical blood relative of patient aged 18-65

Written informed consent.

Meets screening criteria:
Clinical studies will include chest X-ray, FBC with differential and platelets, PT/PTT, and blood chemistries (compete biochemical profile, glucose, and electrolytes). Major erythrocyte antigens, e.g. ABO and Rh systems, will be phenotyped. Serologic testing for transmissible disease will be performed per standard blood banking guidelines for organ and tissue donors including, but are not necessarily limited to hepatitis B and C, HIV, CMV, VDRL, H.zoster, H.simplex, EBV, and toxoplasmosis. Urine or blood pregnancy test negative.

Donor exclusion criteria:

Medically unfit to tolerate peripheral blood apheresis.

Pregnant or lactating women.

Positive serology for HIV1,2, EBV, hepatitis B or C

Psychiatric, addictive, or any disorder which compromises ability to give true informed consent for participation in this study.

History of malignant disease other than non-melanotic localised skin carcinoma/cervical, vaginal in situ neoplasia or current malignancy.

Subject withdrawal criteria

Refusal of donor to participate or unsuitability of donor through failure to meet selection criteria or lack of TaNK generating capacity
Intolerance of conditioning therapy
Failure of TaNK product to meet release criteria
Infusional toxicity during administration of IMP
Patient choice to withdraw

In all cases of patient withdrawal the CI must be informed who will then notify the co-investigator. The reason for withdrawal must be recorded in the patient's CRF. If the patient withdraws or is withdrawn prior to production of his/her specific IMP then another patient may be enrolled in their place and the original patient will not be monitored. If the IMP has been produced it must be discarded as clinical waste. Patients withdrawn during conditioning chemotherapy will not receive the IMP (which will be discarded) but will continue to be monitored as if they had received the IMP. Patients who withdraw during administration of the IMP will continue to be monitored as if they had received the full dose of IMP.

6. Treatment of subjects & donors

Registration:

Patients who meet the eligibility criteria will be approached at the time of their re-induction chemotherapy and the protocol will be discussed with them by the CI. Patients will be given a Patient Information Sheet and have at least 24 hours to decide whether to participate. Subjects wishing to proceed will then be asked to sign the patient consent form and a trial patient Study Number will be assigned from a list pre-supplied to the PI.

The PI will then ensure that appropriate blood/urine samples are taken for the screening tests described in ANNEX 1 and will ask the subject for contact details of potential related donors.

The PI will arrange out-patient appointments for the potential related donors during which he will go through the donor information sheet and request donor consent for participation. The donor will be identified by the same study number as the patient plus a suffix "D-number". The first potential donor for an individual subject will be numbered "1" and subsequent potential donors will be numbered sequentially. EG – the first enrolled subject will be TaNK CTI 001. The first potential donor to be screened for this subject will be identified as TaNK CTI 001-D-1. Each potential donor will then have blood/urine samples taken for the screening tests described in ANNEX 1.

When the PI receives the screening results from the subject and donor(s) he will review them and determine whether an appropriate subject and donor pairing exists.

The subject and donor(s) will be informed of the outcome and a schedule for the treatment will be established. The treatment schedule will be determined by:

a. Donors MUST undergo the apheresis procedure within 30 days of the infectious disease marker testing.
b. The TaNK IMP cannot be released until 16 days after donor apheresis.
c. The subject cannot receive the TaNK IMP until completion of 5 days of chemotherapy and a single fraction TBI.

Donor apheresis:

On at least day -16 prior to planned completion of chemotherapy by the patient the donor will attend the Haematology Day Ward – OACS and undergo a single scheduled 2 hour continuous apheresis session to obtain 5×10^9 mononuclear cells after which (s)he will be free to leave and his/her participation in the trial is complete.

The aphresate will be transferred to the Paul O'Gorman LCT where the mononuclear cells will be stimulated overnight with CTV-1 cell lysate in sterile media. After overnight incubation the NK cell fraction will be isolated by clinical-grade immunomagnetic selection and the resultant product will be enumerated for NK cell content, sampled for sterility and functional testing and cryopreserved as a single patient-specific dose. Upon confirmation of the sterility and function of the TaNK the patient will commence chemotherapy and radiotherapy conditioning as described below. Upon completion of the conditioning regimen the patient-specific IMP will be infused into the patient as described above.

Patient HPC-A collection and pre-infusion conditioning therapy:

Patients who are enrolled will be assessed by the PI for clinical suitability for HPC-A mobilisation and storage. Suitability for HPC-A collection is not a pre-requisite for trial enrolment but, suitable patients will receive 10mg/kg G-CSF per day for 5 days and will then undergo a standard apheresis procedure for the collection of autologous HPC-A which will be cryopreserved. Pre-infusion conditioning chemotherapy will commence at least one week after HPC-A collection.

On Day -3 (Day 0 being the day of TaNK infusion) consented patients will be admitted onto the Haematology In-Patient ward for insertion of a Hickman Line under anaesthetic to facilitate the administration of the chemotherapy. All patients will receive Fludarabine $25mg/m^2$/day on each of the following three days (administered IV over a 30 minute infusion) plus a single fraction (2Gy) TBI on the final day.

Patient infusion with the IMP:

On Day 0 each patient will receive a single "slow push" i.v. infusion of donor TaNK in a 20ml volume. Approximately 10min prior to release of the product (i.e. 30min prior to planned infusion), the patient should receive pre-medication of chlorpheniramine IV and paracetemol. All patients will be monitored for at least 6 hours post infusion before discharge.

Justification of period of hospitalisation:

This represents a treatment schedule of 7 days which is one day longer than that which the patients would have undergone in receipt of standard therapy.

Patients who respond to TaNK but who relapse within 2 years after the TaNK cell infusion

Patients who relapse within 24 months of the TaNK cell infusion and who remain ineligible for any routine treatment might benefit from a second TaNK infusion at the same dose. In some cases sufficient donor TaNK will remain cryopreserved from the original collection and, if a matched pilot vial from the orginal collection is available and passes the release criteria then the associated product can be released for infusion. Alternatively, a second lymphocyte apheresis might be considered from the original donor or a fresh apheresate from their sibling for production of a second product.
Patients will be ineligible for a second infusion if they experienced any product-related SAE within 28 days of the first TaNK infusion.
Patients must be considered fit to undergo a second treatment with TaNK cells.
Data from the first 4 patients treated demonstrate that allogeneic donor NK cells remain at low but detectable levels in recipients' blood for a prolonged period after infusion. This suggests that immunosupression prior to the administration of the second TaNK dose might not be necessary. However, if subsequent laboratory studies show rejection of the infused cells, another infusion can be considered following 3 days of fludarabine at a dose of 25mg/m2. For patients receiving fludarabine the NK cells should be infused no sooner than 48 hours from the last administration of the 3rd dose of fludarabine.
The majority of patients who might be eligible for a second TaNK cell infusion are likely to have overt leukaemia with increased number of blast cells either in the marrow or in the peripheral blood. These patients will require some form of cytoreduction prior the TaNK cell administration; however remission or good partial remission is not a prerequisite for consideration of a second dose. The PI will decide and administer either monotherapy or a combination of cytotoxic chemotherapy and TaNK aiming to reduce the leukaemic load.

Patient on-study evaluations – see Annex 2
Laboratory evaluations:

- Pre-TaNK infusion – 1x 10ml clotted blood sample and 1 x20ml heparinised blood sample will be taken immediately prior to infusion of the donor TaNK product for assessment of baseline anti-CTV1 antibodies and NK chimerism.
- Weekly FBC with differential and platelet count for the first two months followed by fortnightly measurements of the same during months 3 and 4 and monthly assessments in months 5 and 6.
- Serum chemistry profile to include electrolytes, creatinine, urea, SGOT, SGPT, alkaline phosphatase and total bilirubin will follow the same protocol.
- Peripheral blood immune reconstitution and chimerism (T cell subset and NK cell) from one 3ml EDTA sample with matched FBC and lymphocyte differential. Samples will be taken weekly for the first month and then monthly until six months.
- On or around day +30, day +60 day +90, DAY +180 one 20ml heparinised peripheral blood for functional lymphocyte testing (NK cell cytotoxicity in vitro) and one 10ml clotted sample. These are additional samples taken to monitor the impact of the donor NK cell infusion. These samples will be passed to the BMT/Immunotherapy Research Labs, Department of Haematology, Royal Free Hospital. Samples will be tested within 24hr of receipt and aliquots of mononuclear cells will be cryopreserved to allow repeat testing in the case of technical failures. All cryopreserved samples will be discarded within 6 months of completion of the study. At the same time points a single 10ml clotted blood sample will be taken for testing for serum antibodies (IgM and IgG) to CTV-1a

cells to investigate whether any of the CTV-1a cell lysate material was inadvertently administered to the patients as an undetectable contaminant within the TaNK product and whether the lysate is immunogenic. These assays will be conducted at completion of the trial. The generation of antibodies reactive to CTV-1 will be regarded as an “Adverse Event” and recorded in the CRF accordingly. The presence of such antibodies will indicate that the TaNK product was contaminated with CTV-1 lysate. This would not be regarded as a Serious Adverse Event since the lysate is known to be sterile and patient will not be exposed to CTV-1 in the future.

Patient follow-up evaluations (6 months/1year)

- Survival
- Infections
- Full Blood counts and chemistry as described above
- T cell and NK cell profile (to 6 months only)
- NK cell chimerism and function (to 6 months only)
- Anti-CTV-1 antibodies in serum

Special evaluations throughout the study

Evaluation of bone marrow suppression

- **Definitions**
  a. Neutrophil recovery will be defined as the first two consecutive days on which $\geq 0.5 \times 10^9$ neutrophils/l are observed.

  b. Platelet recovery will be defined as the first day that no other platelet transfusions were given for at least 7 days after the first and subsequent platelet transfusions.

  c. Bone Marrow Suppression is defined as a delay in neutrophil and/or platelet recovery by >5 days compared to the median of historical control patients after conventional chemotherapy but not receiving AML TaNK therapy.

  d. Aplastic anaemia is defined as unexpected and inexplicable PMN $<0.5 \times 10^9$/l with Hb <8g and platelets <20 of greater than 28 days.

7. Assessment of efficacy

This is a phase I trial so it is not powered to assess efficacy. However, evidence of engraftment of donor NK cells will be sought by measurement of chimerism status in the NK and non-NK peripheral blood lymphocyte fractions from patients at multiple time points after infusion. Additionally, relapse-free survival and overall survival will be monitored and can be compared with historical controls.

8. Assessment of safety

General monitoring

Concomitant medication will be monitored according the current standard operating procedures in our unit and will include:

- RBC and platelet transfusions
- Antibiotics, anti-fungals, cytokines

Patients will be monitored at least daily as in patients and at each subsequent out patient clinical attendance in accordance with NCI criteria and our own standard operating procedures for:

- Infections
- Bleeding episodes
- Adverse events which are to be recorded as they occur
- Survival status at hospital discharge and at day 100
- Disease status at hospital discharge and at day 100
- Number of days of hospitalization
- Re-hospitalization records
- Pregnancy – instances of pregnancy will be reported immediately to the Sponsor (the PI will submit a form supplied by the Sponsor) and the patient will be monitored until parturition and the baby will be examined for evidence of abnormality.

Definition of Various Adverse Events

In order to comply with the standards for Good Clinical Practice it is essential that investigators are aware of the different definitions related to adverse events and how to record, report and review each of these specific occurrences. For the purpose of this protocol adverse events are classified in to the following categories:

Adverse Event (AE)

AE means any untoward medical occurrence in a clinical trial subject to whom an intervention or medicinal product has been administered, including occurrences which are not necessarily caused by or related to that product or intervention. This includes abnormal laboratory findings, symptoms or disease temporally associated with the use of a medicinal product, whether or not related to the product or intervention.

Adverse Reaction (AR)

AR means any untoward and unintended response in a subject to any intervention or investigational medicinal product, which is related to that intervention or product.

Serious Adverse Event (SAE), Serious Adverse Reaction (SAR), or Unexpected Serious Adverse Reaction

SAE means an adverse event, adverse reaction or unexpected adverse reaction respectively that does not necessarily have a causal relation ship to the treatment, and that at any dose:

- results in death
- is life threatening
- requires hospitalisation or prolongation of existing hospitalisation
- results in persistent or significant disability or incapacity or
- consists of a congenital anomaly or birth defect

Life threatening in the definition of a serious AE or AR refers to an event in which the patient *was at risk of death at the time of event*; it does not refer to an event, which hypothetically might have caused death if it were more severe.

***Suspected <u>Unexpected</u>* Serious Adverse Reaction (SUSAR)**
SUSAR means an adverse reaction that is classed in nature as serious and which <u>is not consistent</u> with the information about the medicinal product or intervention in question set out

- in the case of a licensed product, in the summary of product characteristics (SmPC) for that product
- in the case of any other investigational medicinal product, in the IB relating to the trial in question

Therefore a serious event or drug reaction is not defined as a SUSAR when:

- it is serious but expected
- it does not fit the definition of a SAE or SAR, whether expected or not

Assessment of Causality
The relationship of adverse events to a medicinal product should be determined according to the following classification.

Not Related: the adverse event is not reasonably related to the medicinal product / intervention - or another cause can itself explain the occurrence of the event

Unlikely Related: the adverse event is doubtfully related to the medicinal product / intervention but can't be fully ruled out.

Possibly Related: the adverse event is reasonably related to the medicinal product / intervention, but the event could have been due to another, equally likely cause

Probably Related: the adverse event is reasonably related to the medicinal product / intervention, and the event is more likely explained by the drug than by any other cause

Definitely Related: the adverse event is clearly related to the medicinal product / intervention and there is no other cause to explain the event or a re-challenge (if feasible) is positive

All adverse events, whether or not considered related to the medicinal product or intervention, must be documented in the CRF.

Reporting of Serious Adverse Events (SAEs)
<u>All</u> events that fall into the SAE category except those which are expected as a routine consequence of the standard treatment as defined in Table 1 must be reported (in as much detail as possible) to the SPONSOR by telephone or by fax, **<u>within 24 hours</u>** of observing or learning of the event. This notification must be followed-up in writing using the SAE report form (a copy can be found at the end of this protocol) **<u>within maximum 2 days</u>** (for contact details see above). Medical terminology should always be used to describe any

event. Investigators should avoid vague terms such as "sick". Where some information (e.g. date of resolution of event) is not available at the time of notification, the SAO should be notified of the event within the timelines above and supplementary information provided when available.

Using forms supplied by the Sponsor the following attributes must be assigned when reporting:

- Patient information
- IMP information
- Detailed description of the event
- Dates of onset and resolution
- Severity of the event
- Assessment of relatedness to treatment (see Section 15.3)
- Other suspect drugs/devices
- Action taken and outcome

The investigator may be asked to provide further information, and where a death has occurred autopsy reports and relevant medical reports should be sent with the notification or as soon as available.

The SPONSOR will review all SAEs and classify them as SUSARs or non-SUSARs.

Patients experiencing serious adverse events considered 'related' to the study treatment will be followed up by the Chief Investigator until the event is resolved or considered stable. It will be left to the Chief Investigator's clinical judgment whether or not an adverse event is of sufficient severity to require that the patient should be withdrawn from study treatment. Patients may also voluntarily withdraw from the study due to what he or she perceives as an intolerable adverse event. All patient withdrawals due to adverse events should be documented and reported to the SPONSOR without delay, and patients must be given appropriate care under medical supervision until adverse event symptoms cease or the condition becomes stable.
Any pregnancy occurring during the clinical study should be documented in the CRF and reported to the SPONSOR, and withdrawal of the patient should be considered.

Expedited reporting of SUSARs
As of May 1st 2004, the sponsors (or their representatives) of clinical trials conducted in the EU and EEA must ensure that all relevant information regarding suspected unexpected serious adverse reactions (SUSAR) are recorded and reported in an expedited fashion.

It is a legal requirement of the sponsor to report *fatal or life-threatening SUSARs* within 7 calendar days to the relevant Regulatory Authorities after receiving first notification of the event. Non-fatal and non life-threatening SUSARs must be reported to the Regulatory Authorities within 15 calendar days. It will be the responsibility of the SPONSOR to review all reported SAE's and evaluate them for expectedness, and to report SUSARs to the relevant Regulatory Authorities, Ethics Committees, and institutional bodies as required.

Table 1 – expected adverse events

G-CSF – bone pain, neutrophilia

Apheresis –

Some local bruising. Very occasionally donors feel light headed or feint during the procedure but this is perfectly normal.

Fludarabine –

Immediate: nausea, vomiting, fever chills, fatigue, myelosuppression/pancytopaenia, autoimmune haemolytic anaemia, pulmonary toxicity, cardiovascular oedema, haemolytic cystitis, skin rashes.

Delayed: opportunistic infections subsequent to immune suppression, peripheral neuropathy, weakness, agitation, confusion, visual disturbances.

TBI –

Immediate: myelosuppression/pancytopaenia, nausea, vomiting, fatigue, skin erythema, mucocytis, diarrhoea.

Delayed: Skin pigmentation, secondary cancers, endocrine insufficiencies, lung damage.

NB – Aplastic anaemia is defined as "pancytopaenia with no obvious cause" and is <u>not</u> an expected adverse event.

Allogeneic lymphocyte (TaNK) infusions –

Immediate: erythema at site of infusion, mild fever responsive to analgesics.

Delayed: Mild bruising at site of needle insertion.

Chlorpheniramine IV –

Immediate: sedation, dry mouth, blurred vision, psychomotor impairment, gastrointestinal disturbance, transient hypotension, convulsions (rare).

Delayed: none.

Paracetamol –

Immediate: None.

Delayed: None.

Blood sampling -

Immediate: erythema at site of needle insertion.

Delayed: Mild bruising at site of needle insertion.

Disease-related –

All patients enrolled in this study will have a diagnosis of Acute Myeloid Leukaemia and are therefore at risk of:

Infections, bone marrow failure, neutropaenia and lymphopaenia, bleeding episodes due to low platelet counts, disease relapse or progression.

9. Statistics

This is a phase I safety study and no previous data are available to determine the likely incidence of adverse events following infusion of this particular cell type.

The incidence of each adverse event will be recorded but, there being no control group, this will not be formally tested for statistical significance. The surrogate efficacy markers, NK chimerism and TaNK function, will be analysed upon completion of the trial with respect to the three IMP dose levels. Additionally, disease-free survival and overall survival of the entire study population will be compared with historical controls. The groups will consist of a maximum of 5 subjects so we do not expect to achieve statistically significant differences between the three dose levels.

10. Direct access to source documents

Full and frank disclosure of all results will be made to the SPONSOR and to the relevant regulatory authorities (HTA, MHRA) as required. Academically significant results will be compiled into on or more manuscripts for submission to appropriate scientific journals and/or meetings for dissemination to the wider medical and scientific communities. Patient and donor confidentiality will be maintained throughout.

11. Quality Control & Quality Assurance

The trial will be conducted in compliance with the protocol and with GCP and other relevant regulatory requirements including the EU Tissues & Cells Directives and the Human Tissue Act. The trial will be monitored in accordance with the SPONSOR'S SOPs. As this is a Phase I trial, **all** CRFs will be monitored by the SPONSOR.

12. Ethics

The declaration of Helsinki (App.J.) and its subsequent amendments shall be the accepted basis for the ethical conduct of the clinical investigation. It shall be applied by all parties involved and at every step in the clinical investigation from the first recognition of need and justification to the publication of results.

Informed Consent

Patients are referred for consideration for study registration. The patient's history and current status are completely evaluated, and the course of therapy is decided by the treatment team. These recommendations are then discussed thoroughly with the patient, donor and family. The TaNK cell procedure, as well as any alternative forms of therapy, are presented as objectively as possible. The risks and hazards of the procedures are explained to the patient and donor. The Investigator shall seek consent only under circumstances that provide the patient with sufficient opportunity to consider whether or not to participate and that minimise the possibility of coercion or undue influence. The information that is given to the patient and donor shall be in language understandable to the patient and donor. No informed consent, whether oral or written, may include any exculpatory language through which the patient or donor are made to waive or appear to waive any of their legal rights, or releases or appears to release the investigator, the institution, or its agents from liability for negligence.

The patient must be able to understand the informed consent form and sign and date it prior to patient enrolment. The patient will then receive a copy of the consent. The patient will be free to withdraw consent to enter the trial at any stage and without prejudice to further treatment.

Ethical Committee approval

The Investigator will obtain approval for the study from his local ethical committee. All changes to the protocol must be reviewed and approved prior to implementation except where necessary to eliminate apparent immediate hazards to human subjects.

Patient confidentiality

The Investigator must ensure that patient anonymity is maintained. On the case report forms or other documents, patients should be identified by their initials and a patient study number only. Documents which are not for submission (e.g. signed informed consent form) should be kept in strict confidence by the investigator.

13. Data Handling & Record Keeping

All data will be retained in secure and safe sites for a minimum of 30 years in compliance with the Human Tissue Act (since the IMP is derived from human cells).

14. Financing and indemnity

The additional costs associated with participation in this trial will be met by a research grant from the Leukaemia Research Fund. Some materials used in the production of the IMP will be subsidised by the manufacturer, Miltenyi Biotec. No patient or donor will be paid for participation in the trial.
UCL insurers will provide indemnity.

15. Publication policy

Full and frank disclosure of all results will be made to the sponsor and to the relevant regulatory authorities (HTA, MHRA) as required. Academically significant results will be compiled into on or more manuscripts for submission to appropriate scientific journals and/or meetings for dissemination to the wider medical and scientific communities. Patient and donor confidentiality will be maintained throughout.

Authors:

Dr Panagiotis Kottaridis, Consultant Haematologist, Honorary Senior Lecturer
Dr Mark W Lowdell, Senior Lecturer, Honorary Consultant Immunologist

2008-10-13

References

1. Lowdell, M.W., Shamim, F., Hamon, M., Macdonald, I.D, Prentice, H.G. (1995) VLA-6 (CDw49f) is an important adhesion molecule in NK cell-mediated cytotoxicity following autologous or allogeneic bone marrow transplantation. Experimental Hematology ;23:1530-1534

2. Lowdell MW, Craston R, Samuel D, Wood ME, O'Neil E, Saha V, Prentice HG. (2002) Evidence that continued remission in patients treated for acute leukaemia is dependent upon autologous natural killer cells. Br J Haematology; 117:821-827.

3. Gehrmann M, Schmetzer H, Eissner G, et al. Membrane-bound heat shock protein 70 (HSP70) in acute myeloid leukemia: a tumor specific recognition structure for the cytolytic activity of autologous NK cells. Haematologica. 2003;88:474-6.

4. Ruggeri L et al Effectiveness of donor natural killer cell alloreactivity in HLA mismatched hematopoietic transplants. Science 2002;295: 2097-2100.

5. Giebel S, Locatelli F, Lamparelli T, et al. Survival advantage with KIR ligand incompatibility in hematopoietic stem cell transplantation from unrelated donors. Blood. 2003;102:814-19.

6. Koh CY, Ortaldo JR, Blazer BR et al. NK cell purging of leukaemia: superior effects of NK cells H-2 allogeneic to the leukemia and augmentation with inhibitory receptor blockade. Blood 2003; 102: 4067-

7. Miller JS, Soignier Y, Panoskaltsis-Mortari et al. Successful adoptive transfer and in vivo expansion of human haploidentical NK cells in patients with cancer. Blood 2005;105(8):3051-7.

8. North J, Bakhsh I, Marden C, Pittman H, Addison E, Navarrete C, Anderson R, Lowdell MW. (2007) Tumor-primed human natural killer cells lyse NK-resistant tumor targets: evidence for a two-stage process in resting NK cell activation. Journal of Immunology; 178:85-94.

ANNEX 1

Pre-commencement patient and donor screening

Rationnale:

Patients and donors must be evaluated to assess compliance with the inclusion/exclusion criteria through the following laboratory tests:

Subject	Donor
	TaNK activity in vitro<br>1x20ml PFH blood sample
Infectious disease marker screening:<br>HIV 1+2 – anti-HIV1 & 2<br>HepB – HbsAg, anti-HBc<br>HepC – anti-HCV<br>EBV – anti-EBV<br>Syphilis – anti-syphilis antibody<br>2x10ml clotted blood samples	Infectious disease marker screening:<br>HIV 1+2 – anti-HIV1 & 2<br>HepB – HbsAg, anti-HBc<br>HepC – anti-HCV<br>EBV – anti-EBV<br>Syphilis – anti-syphilis antibody<br>2x10ml clotted blood samples
Pregnancy test (women with child bearing potential only)<br>MSU or 1x5ml clotted blood sample	Pregnancy test (women with child bearing potential only)<br>MSU or 1x5ml clotted blood sample

ANNEX 2

TaNK in AML Trial - SUMMARY

1. Patient assessment post completion of chemotherapy Eligible, suitable, consent
2. Patient enrolment – OBTAIN UPN FROM CLINICAL TRIALS OFFICE x35473
3. Donor assessment and consent for testing and for donation Eligible, suitable, consent, IDM –ve, can generate TaNK in vitro.
4. Donor enrolment – ENROLMENT FAILURE – CONTACT CTO FOR REMOVAL OF PATIENT FROM THE TRIAL
5. Donor apheresis
6. TaNK generation and QC
7. CHECK – IMP passes QC – IMP FAILURE – CONTACT CTO FOR REMOVAL OF PATIENT FROM TRIAL
8. Day -5 Patient can commence conditioning chemotherapy
9. Day -1 Patient receives radiotherapy
10. Day 0 Patient pre-infusion blood tests
    a. FBC/Diff
    b. Serum chemistry profile
    c. Immune phenotyping and chimerism
    d. Serum sample storage for anti-CTV-1 antibody testing
11. Day 0 Infusion of IMP
12. Day +7 (approximately)
    a. FBC/Diff
    b. Serum chemistry profile
    c. Immune phenotyping and chimerism
13. Day +14 (approximately)
    a. FBC/Diff
    b. Serum chemistry profile
    c. Immune phenotyping and chimerism
14. Day +21 (approximately)
    a. FBC/Diff
    b. Serum chemistry profile
    c. Immune phenotyping and chimerism
15. Day +28 (approximately) – 1 month
    a. FBC/Diff
    b. Serum chemistry profile
    c. Immune phenotyping and chimerism
    d. 20ml heparinised blood for TaNK function
    e. Serum sample storage for anti-CTV-1 antibody testing
16. Day +35 (approximately)
    a. FBC/Diff
    b. Serum chemistry profile
17. Day +42 (approximately)
    a. FBC/Diff
    b. Serum chemistry profile
18. Day +49 (approximately)
    a. FBC/Diff
    b. Serum chemistry profile
19. Day +56 (approximately) - 2 months
    a. FBC/Diff
    b. Serum chemistry profile

c. Immune phenotyping and chimerism

d. 20ml heparinised blood for TaNK function

e. Serum sample storage for anti-CTV-1 antibody testing

20. Day +70 (approximately)

    a. FBC/Diff

    b. Serum chemistry profile

21. Day +84 (approximately) – 3 months

    a. FBC/Diff

    b. Serum chemistry profile

    c. Immune phenotyping and chimerism

22. Day +98 (approximately)

    a. FBC/Diff

    b. Serum chemistry profile

    c. 20ml heparinised blood for TaNK function

    d. Serum sample storage for anti-CTV-1 antibody testing

23. Day +112 (approximately) – 4 months

    a. FBC/Diff

    b. Serum chemistry profile

    c. Immune phenotyping and chimerism

24. Day +140 (approximately) – 5 months

    a. FBC/Diff

    b. Serum chemistry profile

    c. Immune phenotyping and chimerism

25. Day +170 (approximately) – 6 months

    a. FBC/Diff

    b. Serum chemistry profile

    c. Immune phenotyping and chimerism

    d. 20ml heparinised blood for TaNK function

    e. Serum sample storage for anti-CTV-1 antibody testing
